# *NFκB1* is a suppressor of neutrophil-driven hepatocellular carcinoma

**DOI:** 10.1038/ncomms7818

**Published:** 2015-04-16

**Authors:** C. L. Wilson, D. Jurk, N. Fullard, P. Banks, A. Page, S. Luli, A. M. Elsharkawy, R. G. Gieling, J. Bagchi Chakraborty, C. Fox, C. Richardson, K. Callaghan, G. E. Blair, N. Fox, A. Lagnado, J. F. Passos, A. J. Moore, G. R. Smith, D. G. Tiniakos, J. Mann, F. Oakley, D. A. Mann

**Affiliations:** 1 Fibrosis Research Group, Institute of Cellular Medicine, Newcastle University, Newcastle upon Tyne NE2 4HH, UK; 2 Newcastle University Institute for Ageing and Institute for Cell and Molecular Biosciences, Newcastle University, Newcastle Upon Tyne NE4 5PL, UK; 3 Liver Unit, University Hospitals Birmingham, Birmingham B15 2TH, UK; 4 Hypoxia and Therapeutics Group, Manchester Pharmacy School, University of Manchester, Manchester M13 9PT, UK; 5 Department of Medicine, Immunology and Inflammation, Imperial College of Science, Technology and Medicine, Hammersmith Hospital, London W12 0NN, UK; 6 Centre for Behaviour and Evolution/Institute of Neuroscience, Medical School, Newcastle University, Newcastle Upon Tyne NE2 4HH, UK; 7 Faculty of Biological Sciences, School of Molecular and Cellular Biology, University of Leeds, Garstang Building, Leeds LS2 9JT, UK; 8 Institute for Cell and Molecular Biosciences, Newcastle University, Catherine Cookson Building, Framlington Place, Newcastle Upon Tyne NE2 4HH, UK

## Abstract

Hepatocellular carcinoma (HCC) develops on the background of chronic hepatitis. Leukocytes found within the HCC microenvironment are implicated as regulators of tumour growth. We show that diethylnitrosamine (DEN)-induced murine HCC is attenuated by antibody-mediated depletion of hepatic neutrophils, the latter stimulating hepatocellular ROS and telomere DNA damage. We additionally report a previously unappreciated tumour suppressor function for hepatocellular *nfkb1* operating via p50:p50 dimers and the co-repressor HDAC1. These anti-inflammatory proteins combine to transcriptionally repress hepatic expression of a S100A8/9, CXCL1 and CXCL2 neutrophil chemokine network. Loss of *nfkb1* promotes ageing-associated chronic liver disease (CLD), characterized by steatosis, neutrophillia, fibrosis, hepatocyte telomere damage and HCC. *Nfkb1*^*S340A/S340A*^mice carrying a mutation designed to selectively disrupt p50:p50:HDAC1 complexes are more susceptible to HCC; by contrast, mice lacking S100A9 express reduced neutrophil chemokines and are protected from HCC. Inhibiting neutrophil accumulation in CLD or targeting their tumour-promoting activities may offer therapeutic opportunities in HCC.

## Abstract (web summary)

The role of neutrophils in cancer development is not widely appreciated. Here, the authors show that NF-κB-deficient hepatocytes overproduce chemokines, leading to hepatocellular carcinoma due to excessive neutrophil recruitment, and that neutrophil depletion prevents liver cancer in these mice.

## Graphical abstract

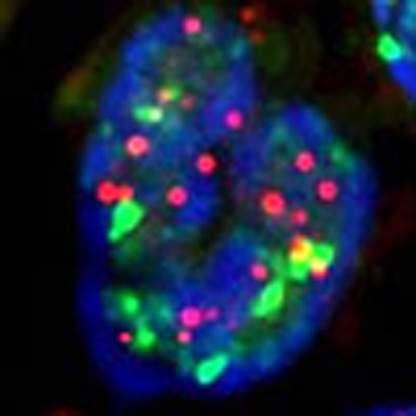

The mechanisms driving the progression of chronic inflammatory liver diseases such as alcoholic steatohepatitis and non-alcoholic steatohepatitis (ASH and NASH) to hepatocellular carcinoma (HCC) are poorly understood. The majority of HCC develops on the background of cirrhosis^1^, which is the end-stage result of fibrogenesis, a process of excessive wound repair. This has led to the concept that the fibrotic extracellular matrix and/or pro-fibrogenic myofibroblasts may be stimulators of HCC^2^. However, the increasing awareness that non-cirrhotic HCC represents a high proportion of liver cancers argues for additional mechanisms inherent in the inflammatory process. Inflammation underpins roughly 20% of all solid tumours and experimental studies have provided mechanistic links between inflammatory mediators such as interleukin (IL)-6 and carcinogenesis^3^. The histopathological changes associated with chronic ASH and NASH include persistent tissue infiltration by neutrophils and lymphocytes^4^. Appearance of neutrophils in the hepatic parenchyma is normally a transient process triggered by damage-induced expression of soluble and insoluble neutrophil guidance cues and is subsequently terminated by neutrophil death and/or clearance by Kupffer cells^5^,^6^. This transitory response is important for resolution of inflammation and for protecting epithelial cells from bystander damage resulting from exposure to neutrophil-derived reactive oxygen species (ROS) and proteases. Persistence of neutrophils in chronic liver disease (CLD) is therefore a pathological feature; however, it remains unclear as to how neutrophils contribute to disease progression. Experimental work in rodent models argues against a role for neutrophils in wound repair or fibrogenesis^7^,^8^. As neutrophils are present in human HCC and its surrounding tissue (Supplementary Fig. 1a), we were interested to determine whether they functionally contribute to tumour development. Here we demonstrate a requirement of hepatic neutrophils for the development of carcinogen-induced HCC and define a pro-tumour mechanism in which neutrophils cause telomere DNA damage in bystander hepatocytes. We additionally define a tumour suppressor role for the *nfkb1* gene, which through the expression of anti-inflammatory p50:p50 nuclear factor-κB (NF-κB) dimers is able to transcriptionally repress a neutrophil chemokine network.

## Results

### Neutrophils are required for experimentally induced HCC

**Figure 1:**
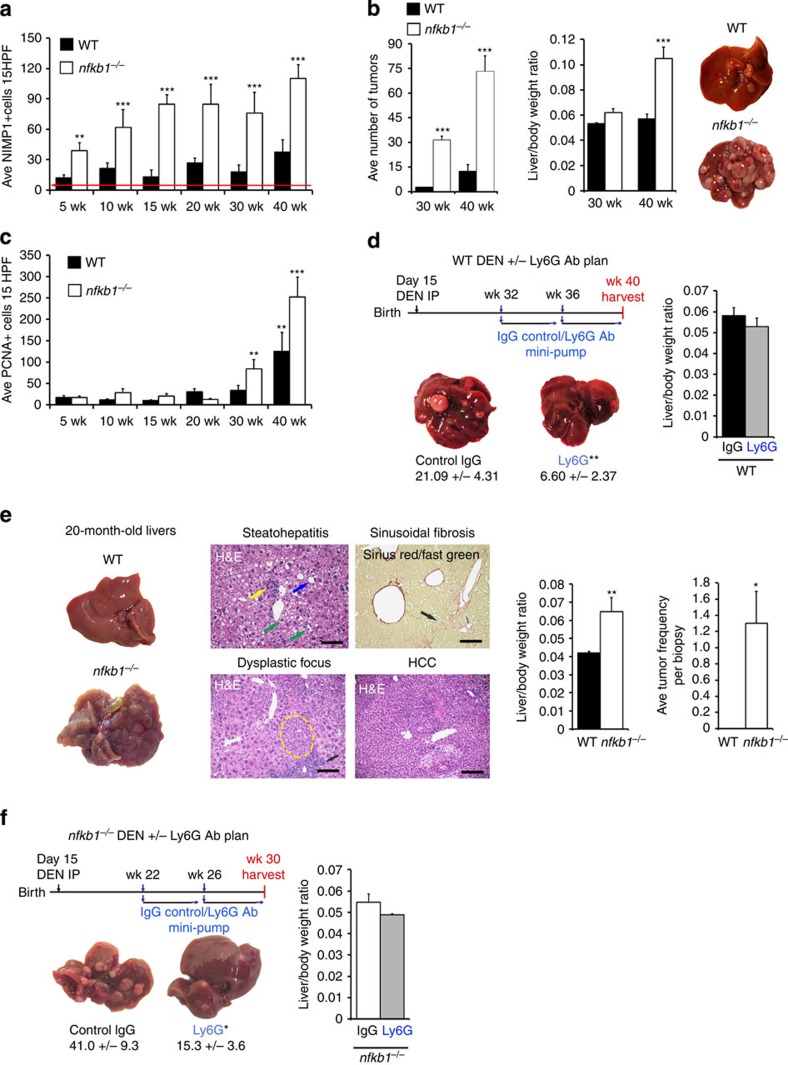
Neutrophils are required for experimentally induced HCC. (**a**) Graph shows average total number of hepatic neutrophils ( NIMP-R14 ) in DEN-injured WT or *nfkb1^−/−^* mice. Red line denotes basal neutrophil levels in normal WT liver. (**b**) Average tumour counts and liver/body weight ratio in 30- and 40-week DEN-injured WT and *nfkb1^−/−^* mice. Representative pictures of livers from 40-week DEN-injured WT and *nfkb1^−/−^* mice. (**c**) Average total number of PCNA+ hepatocytes in DEN-injured WT and *nfkb1^−/−^* mice. (**d**) Diagram showing the experimental plan to deplete neutrophils (Ly6G antibody) in WT mice from week 32–40 after DEN injury. Average tumour counts, representative pictures of livers and average liver/body weight ratio graph in 40-week DEN-injured WT mice treated with control IgG or Ly6G antibody (Ab) for 8 weeks. *n*=10 (**e**) Representative pictures of liver from 20 month (aged under normal conditions) WT and *nfkb1^−/−^* mice. Representative photomicrographs of haematoxylin and eosin (H&E)- and Sirius red/fast green-stained liver sections from 20-month *nfkb1^−/−^* mice (upper panel) revealed steatohepatitis (fat, blue arrows; inflammation, yellow arrows; ballooned hepatocytes with Mallory–Denk bodies, green arrows) and fibrosis (black arrows). Focal dysplasia (yellow dotted line) with focal inflammation (black arrow) and HCC in H&E-stained *nfkb1^−/−^* aged livers (lower panel). Graphs show average liver/body weight ratio and tumour frequency identified histologically using H&E sections from 20-month, aged WT and *nfkb1^−/−^* mice. *n*=7 WT and ten *nfkb1^−/−^* mice. (**f**) Diagram showing the neutrophil depletion experimental protocol in *nfkb1^−/−^* mice from week 22–30 after DEN injury. Average tumour counts, representative pictures of livers and graph showing average liver/body weight ratio from 30-week DEN-injured *nfkb1^−/−^* mice treated with control IgG or Ly6G for 8 weeks (*n*=10). All data are means±s.e.m; scale bars, 200 μm. For **a** and **c**, *n*=6, 4, 6, 7, 15 and 9 (*nfkb1^−/−^*), and 4, 4, 4, 5, 11 and 14 (WT) for the 5- to 40-week time points). Statistical significance was determined using one-way analysis of variance with Tukey's *post-hoc* test (**a**,**c**) or an unpaired *t*-test (**b**,**e**), *P<0.05, **P<0.01 or ***P<0.001 compared with control.

Diethylnitrosamine (DEN) acts as a complete liver carcinogen when administered to mice 15 days after birth^9^. However, a role for inflammatory mechanisms in this model is demonstrated by the requirement for NF-κB activation in myeloid cells and for the production of IL-6 by Kupffer cells, for injury to progress to HCC^10^,^11^. To begin to investigate a role for neutrophils in this process, we examined hepatic neutrophils across a 5- to 40-week time course of DEN-induced disease in male C57BL/6 mice (Fig. 1a). Elevated numbers of hepatic neutrophils were evident throughout the disease process and underwent a steady increase until 40 weeks where they were accompanied by enlarged livers, substantial tumour frequency (Fig. 1b), large tumours (Supplementary Fig. 1b) and high numbers of PCNA+ proliferating hepatocytes (PCNA+) (Fig. 1c). To determine whether neutrophils contribute to DEN-induced HCC, mice were treated between weeks 32 and 40 with an antibody specific for the mouse neutrophil antigen Ly6G, known to effectively suppress neutrophil recruitment to tissues^12^. We confirmed that anti-Ly6G suppressed DEN-induced hepatic neutrophil accumulation with little effect on F4/80+ macrophages or CD3+ lymphocytes (Supplementary Fig. 1c). Selective depletion of neutrophils was associated with a 3.5-fold reduction in tumour burden at 40 weeks and a slight reduction in liver/body weight ratio (Fig. 1d). Mice lacking *nfkb1* (encoding the NF-κB p50 subunit) display exaggerated neutrophil inflammatory responses to injury in multiple organs, including the liver^13^,^14^,^15^. Early (5 weeks) and persistent elevation of hepatic neutrophils was observed in DEN-injured *nfkb1^−/−^* mice (Fig. 1a). Furthermore, these animals exhibited rapid progression to aggressive HCC (Fig. 1b). Of note, Cyp2E1 expression (Supplementary Fig. 1d) and levels of liver injury and apoptosis (Supplementary Table 1 and Supplementary Fig. 1e) were similar between the two genotypes, ruling out the possibility that differences in DEN-induced liver injury caused the increased tumour burden. Dysplastic cells and small tumours were evident at 20 weeks (Supplementary Fig. 2a) in *nfkb1^−/−^* mice, and by 30 weeks there was an average of 32 visible surface liver tumour foci compared with just 3 for wild type (WT; Fig. 1b). By week 40, an average of 73 and 12 surface growths were recorded for *nfkb1^−/−^* and WT, respectively. Accelerated disease in *nfkb1^−/−^* liver was confirmed by increased cell proliferation (Fig. 1c), elevated hepatic expression of cyclin D1 (Supplementary Fig. 2b) and loss of phospho-p38α (Supplementary Fig. 2c), the latter being a feature of advanced human HCCs^16^. C57BL/6 mice have a characteristically low rate (<4%) for the development of spontaneous liver cancer. We were therefore surprised to observe spontaneous CLD, a nodular appearance of the liver surface, emergence of dysplastic nodules, an increase in liver/body weight ratio and tumour frequency at 20 months in *nfkb1^−/−^* males (Fig. 1e). Histological assessment of the livers revealed that *nfkb1^−/−^* mice develop a spectrum of features of CLD, including steatohepatitis, similar to that in humans, lobular mixed inflammation and hepatocyte ballooning occasionally with Mallory–Denk inclusions or megamitochondria, sinusoidal fibrosis and bridging fibrosis in 50% of the mice (Fig. 1e and Supplementary Fig. 2d). In five out of ten mice, hepatocellular adenomas and/or well-to-moderately differentiated HCC were observed (Supplementary Fig. 2e,f) in non-cirrhotic liver. In two cases, portal biliary lesions resembling biliary microhamartomas with no evidence of epithelial dysplasia were noted (Supplementary Fig. 2f). Conversely, old WT mice develop mild steatosis and some focal inflammation (Supplementary Tables 2 and 3). The spontaneous hepatic lesions in aged *nfkb1^−/−^* mice were associated with highly elevated numbers of hepatic neutrophils (Supplementary Fig. 2g). To determine a functional contribution of neutrophils in the *nfkb1^−/−^* background, DEN-injured mice were treated with either the neutrophil-depleting antibody anti-Ly6G or control IgG between weeks 22 and 30 (this being the period of progression from dysplasia to HCC). Anti-Ly6G treatment effectively reduced numbers of hepatic neutrophils, normalized the liver/body weight ratio (Supplementary Fig. 2h and Fig 1f) and blunted tumour development (Fig. 1f) without affecting macrophage or T-lymphocyte recruitment (Supplementary Fig. 2h). Taken together, these data implicate neutrophils as important functional contributors to inflammation-driven HCC.

### Accelerated HCC in the *nfkb1^−/−^* mouse

**Figure 2:**
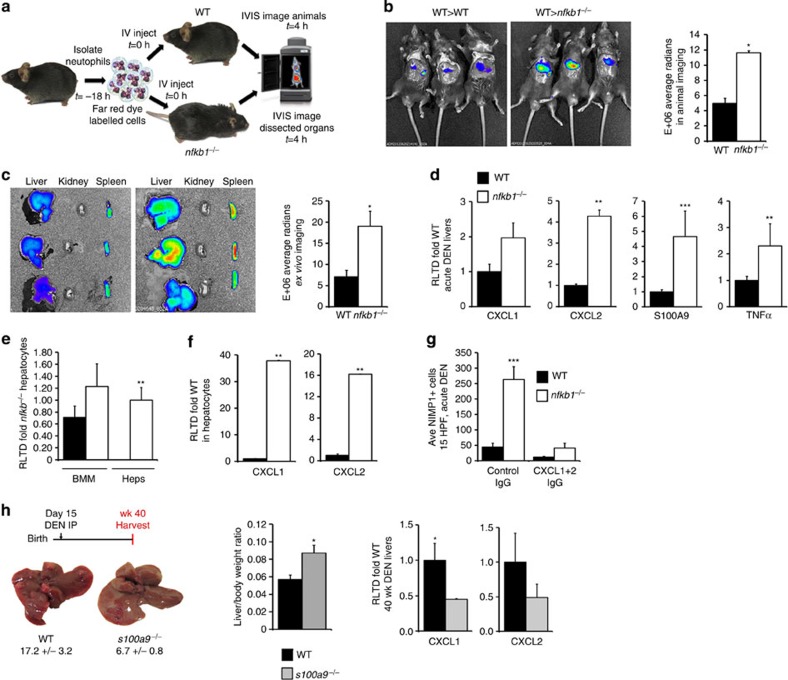
Dysregulation of hepatic chemokine expression and accelerated HCC in the *nfkb1^−/−^* mouse. (**a**) Diagram of the experimental plan using whole-body imaging to track WT neutrophils to the liver of acute DEN-injured WT or *nfkb1^−/−^* mice. (**b**) Representative IVIS pictures of mice given NIR815-labelled WT neutrophils intravenously, showing neutrophils tracking to livers of acute DEN-injured WT or *nfkb1^−/−^* mice. Graph shows average radians from IVIS-imaged WT or *nfkb1^−/−^* mice. (**c**) Representative *ex vivo* images of the liver (left), kidney (middle) and the spleen (right), and graph showing average radians from WT or *nfkb1^−/−^* livers imaged *ex vivo*. *n*=3 (**d**) Hepatic CXCL1, CXCL2, S100A9 and tumour necrosis factor-α (TNFα) mRNA levels expressed as relative level of transcription difference (RLTD) compared with WT 48 h post acute DEN in WT and *nfkb1^−/−^* mice, *n*=6. (**e**) Graph shows RLTD of S100A9 in bone marrow macrophages (BMM) compared with hepatocytes isolated from WT and *nfkb1^−/−^* mice, *n*=3. (**f**) Hepatocyte CXCL1 and CXCL2 mRNA levels expressed as RLTD in *nfkb1^−/−^* mice compared with WT, *n*=3. (**g**) Graph shows average total number of NIMP-R14 cells in liver sections from WT or *nfkb1^−/−^* mice after acute DEN injury-treated±IgG or CXCL1 and two neutralizing antibody, *n*=5. (**h**) Average tumour counts, representative pictures of livers and graph showing average liver/body weight ratio from 40-week DEN-injured WT and *s100a9^−/−^* mice, *n*=19. Hepatic CXCL1 and CXCL2 mRNA levels expressed as RLTD in 40-week, chronic, DEN-injured WT and *s100a9^−/−^*mice, *n*=6. Data are means±s.e.m. Statistical significance was determined using an unpaired *t*-test, *P<0.05, **P<0.01 or ***P<0.001 compared with control.

Cancer models in extra-hepatic organs have suggested pro-tumour functions for neutrophils, and that inhibition of neutrophil recruitment or activities may be therapeutic options in the context of chronic inflammatory disease^17^,^18^,^19^,^20^,^21^. Interrogating the mechanisms responsible for enhanced neutrophil inflammation and aggressive HCC phenotype of the *nfkb1^−/−^* mouse will therefore provide valuable mechanistic insights. Initial investigations ruled out intrinsic differences in neutrophil functions such as apoptosis or production of ROS (Supplementary Fig. 3a,b). We therefore reasoned that either *nfkb1^−/−^* hepatocytes have a deficiency in their control of neutrophil recruitment, or/and *nfkb1^−/−^* neutrophils have an enhanced ability to traffic to the liver. To test the former idea, WT neutrophils were labelled *ex-vivo* with Cellvue NR185 tracker before transfer into the circulation of DEN-injured WT and *nfkb1^−/−^* mice (Fig. 2a). Mice were then imaged 4-h post transfusion by *in vivo* imaging system (IVIS) for hepatic accumulation of labelled neutrophils, which were discovered to be more abundant in *nfkb1^−/−^* compared with WT liver (Fig. 2b). This difference was also observed *ex-vivo* in isolated organs (Fig. 2c) and, of note, labelled neutrophils were restricted to the liver and the spleen. Carrying out the reverse experiment, labelled WT or *nfkb1^−/−^* neutrophils were transferred into DEN-injured WT animals and hepatic accumulation determined by IVIS. No differences were observed between the genotypes, suggesting that the absence of *nfkb1^−/−^* within the neutrophil does not have an intrinsic effect on its migratory ability (Supplementary Fig. 3c). To confirm these data, we produced chimeric mice in which either *nfkb1^−/−^* bone marrow was reconstituted into an irradiated WT background (*nfkb1^−/−^*BM>WT) or WT bone marrow was reconstituted in the *nfkb1^−/−^* background (WTBM>*nfkb1^−/−^*). In response to acute DEN injury, neutrophils were recruited to the liver in both backgrounds, but of note higher numbers of hepatic neutrophils were present in WTBM>*nfkb1^−/−^* mice (Supplementary Fig. 3d). Of note, both the *nfkb1^−/−^*BM>WT and WTBM>*nfkb1^−/−^* animals were considered too sick for use in longer-term studies that would have confirmed the hepatocyte-specific actions of *nfkb1^−/−^* in HCC; this phenotype may be related to our recent report that *nfkb1^−/−^* cells are highly susceptible to radiation-induced senescence^22^. We conclude that enhanced neutrophil recruitment in *nfkb1^−/−^* mice most probably reflects a defect in the hepatic regulation of neutrophil trafficking. We have recently reported that neutrophil migration to the injured liver is critically dependent on a hepatic neutrophil chemokine network comprised of calprotectin (S100A8/S100A9), CXCL1 (Groα/KC) and CXCL2 (Groβ/MIP2), and as the expression of these proteins is under the transcriptional control of NF-κB, it was relevant to determine their expression in *nfkb1^−/−^* liver^7^. CXCL2, S100A9 and tumour necrosis factor-α were all significantly overexpressed in DEN-injured *nfkb1^−/−^* liver (Fig. 2d and Supplementary Table 4). Modest increases for CXCL1, S100A8 and IL-6 were also observed but failed to reach statistical significance (Fig. 2d and Supplementary Fig. 4a). CXCL1, CXCL2, S100A9, IL-6 and tumour necrosis factor-α were also highly upregulated in the diseased livers of 3-, 9-, 12-, 15- and 20-month *nfkb1^−/−^* males (Supplementary Fig. 4b and Supplementary Table 4). At the protein level, S100A9 overexpression was located to mononuclear cells and hepatocytes, and particularly strong S100A9 staining was found within *nfkb1^−/−^* tumour tissue (Supplementary Fig. 4c). Analysis of S100A9 messenger RNA expression in isolated macrophages demonstrated no difference between genotypes; by contrast, S100A9 was not expressed in WT hepatocytes but was detected in *nfkb1^−/−^* hepatocytes (Fig. 2e). Expression of the hepatocyte markers albumin and HNF4α, and macrophage markers F4/80 and CD68 verified the purity of the cell isolations (Supplementary Fig. 4d). CXCL1 and CXCL2 transcripts were highly overexpressed in cultured primary *nfkb1^−/−^* versus WT hepatocytes (Fig. 2f), this being confirmed by enzyme-linked immunosorbent assay (ELISA) for CXCL2 (Supplementary Fig. 4e). Furthermore, number of S100A9+ cells and hepatic expression of S100A9 mRNA were higher in acute DEN-injured WTBM>*nfkb1^−/−^* compared with *nfkb1^−/−^*BM>WT chimeric mice (Supplementary Fig. 3d). These observations confirm a previous report that calprotectin expression is induced in damaged epithelia and HCC cells^23^. To formally establish a requirement of CXCL1 and CXCL2 for migration of neutrophils to DEN-injured liver, we neutralized the chemokines by an antibody approach in the context of acute hepatic injury. Antibody-mediated combined inhibition of CXCL1 and CXCL2 abrogated DEN-induced accumulation of neutrophils in *nfkb1^−/−^* mice (Fig. 2g). Unfortunately, this approach was not practical for determining the role of CXCL1 and CXCL2 in HCC due to the need for longer-term administration of high cost antibodies. However, calprotectin regulates the hepatic expression of both CXCL1 and CXCL2. *s100a9^−/−^* mice express reduced levels of both chemokines upon liver injury^7^ and transgenic expression of S100A8 and S100A9 stimulates overproduction of CXCL1 and mobilization of neutrophils from the bone marrow^24^. Furthermore, neutrophil recruitment is reduced in *s100a9^−/−^* mice in acute DEN injury (Supplementary Fig. 4f). We therefore determined the susceptibility of *s100a9^−/−^* to DEN-induced HCC and discovered that these animals are protected compared with WT. Reduced tumour burden in these animals was associated with reduced hepatic expression of CXCL1 and CXCL2 (Fig. 2h). We conclude that hepatocellular *nfkb1* negatively regulates a neutrophil chemokine network comprising calprotectin, CXCL1 and CXCL2. In support of this network having a pro-tumour function, inhibition of CXCL1 and CXCL2 receptor CXCR2 is sufficient to suppress inflammation-driven skin and intestinal cancers^19^. Furthermore, *s100a9^−/−^* mice are protected against cancers of inflammatory origin, including breast, pancreatic and colitis-associated tumours^25^,^26^,^27^, although loss of S100A9 is associated with susceptibility to experimental skin cancer^28^. To further define the relative contributions of these chemokines to the pathobiology of HCC, it will be necessary to generate conditional genetic deletions enabling temporal control of their expression targeted selectively to hepatocytes.

### Disruption of p50 homodimers increases susceptibility to HCC

**Figure 3:**
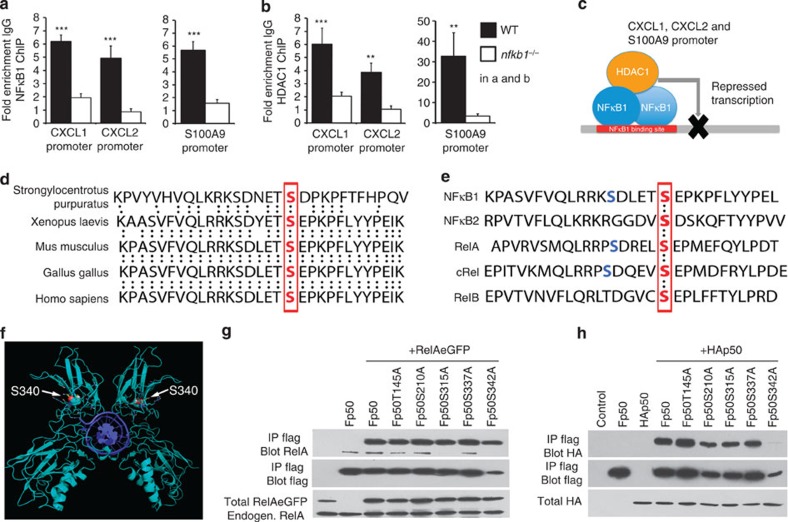
p50 homodimers complexed with HDAC1 regulate the chemokines CXCL1 and 2. (**a**,**b**) ChIP assay analysis of p50 (**a**) and HDAC1 (**b**) recruitment to the CXCL1, CXCL2 and S100A9 promoters in acute DEN-injured WT or *nfkb1−/−* livers. (**c**) Model showing repression of the CXCL1, CXCL2 and S100A9 promoters by p50:p50 homodimers complexed with HDAC1. (**d**) Sequence alignment of *nfkb1* gene shows conservation of amino acid serine 340 (in mouse) throughout evolution from purple sea urchin through to human. The conserved serine is shown in bold and red. (**e**) Sequence alignment of the mouse NF-κB subunits, show conservation of serine 340 (in mouse) throughout all subunits (shown in bold and red) and conservation of serine 337 in NF-κB1, RelA and cRel (shown in bold and blue). (**f**) X-ray crystal structure of the mouse p50:p50 homodimer (cyan) bound to DNA (deep blue), PDB entry 1NFK. White arrows show serine 340, which is rendered in space-filling representation. (**g**) Representative western blots of anti-Flag or anti-RelA after Flag IP on lysates from Cos-7 cells transfected with RelA-eGFP±Flag-p50 or Flag-p50 mutant shows that p50 mutants retain interaction with RelA. (**h**) Western blots for anti-HA or anti-Flag after Flag IP on lysates from Cos-7 cells transfected with Flag-p50 or Flag-p50 mutant±HA-p50 reveals that p50 mutants bind p50, except p50 S342A (human equivalent of mouse 340). Data are means±s.e.m. and representative of five mice per group. Statistical significance was determined using an unpaired *t*-test, **P<0.01 or ***P<0.001 compared with control.

Previous studies propose a model in which the anti-inflammatory properties of *nfkb1* are mediated by p50:p50 homodimers^29^,^30^,^31^,^32^,^33^. Importantly, p50 lacks inherent transcriptional activity and in homodimer form associates with co-repressors such as HDAC1 or Bcl3, to actively repress inflammatory gene transcription^29^,^32^,^34^,^35^. Of note, the nuclei of unstimulated cells contains a significant number of p50:p50 dimers that are assumed to repress transcription^36^. Chromatin immunoprecipitation (ChIP) assays confirmed that p50 and HDAC1 are recruited to the *cxcl1*, *cxcl2* and *s100a9* promoters (Fig. 3a,b). In contrast, HDAC1 did not associate with these promoters in *nfkb1^−/−^* liver (Fig. 3b), supporting a model in which p50:p50 dimers recruit repressive HDAC1 (or/and Bcl3) to suppress hepatic expression of neutrophil chemokines (Fig. 3c). The mechanisms that control NF-κB dimer assembly and, in particular, the choice of heterodimers versus homodimers are very poorly understood, yet will dictate the formation of anti-inflammatory p50:p50:HDAC1 versus pro-inflammatory RelA:p50 complexes. Sequence analysis of p50 protein reveals a region spanning residues 325 to 354 (human p50) displaying high conservation across evolutionarily distant species separated by 743 million years (Fig. 3d). Aligning this region with equivalent sequences in the mammalian NF-κB subunits revealed perfect conservation of two serine residues, Ser337 and Ser342 (Fig. 3e). We noted in the structure of mouse p50:p50 dimers bound to DNA that the equivalent residue to human Ser342 (mouse Ser340) is located on the opposing face from the dimerization interface where it might exert conformational influences on dimer assembly (Fig. 3f). Alanine mutations were introduced at Ser337, Ser342 and at other evolutionary conserved residues (Thr145, Ser210 and Ser315) in the context of human FLAG-p50. WT and mutant Flag-p50 proteins were co-expressed together with HA-p50 or EGFP-RelA, to determine their relative abilities to assemble homodimers or heterodimers, respectively. Pull-down assays demonstrated that all mutant p50 constructs interacted with RelA to a similar degree as WT-p50. Of note, the Ala342 mutant protein was consistently expressed at reduced levels compared with WT-p50 and other mutants (Fig. 3g). Homodimer interactions were similar between WT-p50 and the 145, 210, 315 and 337 alanine mutants (Fig. 3h). In contrast, the Ala342 mutant disabled p50:p50 assembly (Fig. 3h). These mutagenesis experiments therefore reveal the Ser342 residue to be a specific and critical determinant of p50 homodimer assembly. In further experiments, FLAG-Supershift EMSA experiments confirmed that FLAG-p50-Ala342 fails to bind to κB motifs as would be expected, as p50 monomers are unable to bind DNA (Supplementary Fig. 5a). The effects of the mutation on chemokine gene expression were examined by adenoviral overexpression of WT and mutant p50 proteins in mouse hepatocytes (Supplementary Fig. 5b). As anticipated, elevated levels of WT-p50 suppressed expression of CXCL1, but this effect was not observed in cells expressing the mutant homodimerization-defective p50 (Supplementary Fig. 5c). This result confirms that p50:p50 dimers are repressors of neutrophil chemokine expression.

**Figure 4:**
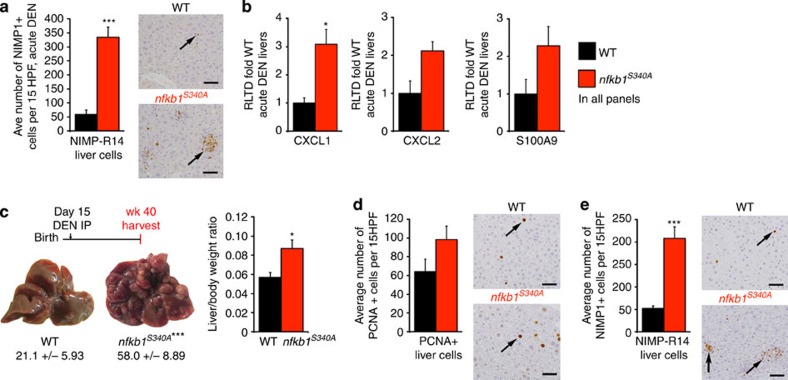
*nfkb1*^*S340A*^ knock-in mice have increased neutrophils and tumour burden in experimentally induced HCC. (**a**) Representative photomicrographs of NIMP-R14 staining and graph showing average total number of neutrophils in liver sections from WT or *nfkb1*^*S340A*^ mice following acute DEN injury. Black arrows denote NIMP-R14 stained cells, *n*=5 (**b**) Hepatic CXCL1, CXCL2 and S100A9 mRNA 48 h post acute DEN in WT and *nfkb1*^*S340A*^ mice. *n*=5 (**c**) Representative pictures of livers, average tumour counts and liver/body weight ratio in 40-week DEN-injured WT and *nfkb1*^*S340A*^ mice. (**d**) Graphs show average total number of PCNA+ stained cells and representative photomicrographs at × 200 magnification of PCNA staining in WT and *nfkb1*^*S340A*^ 40-week DEN-injured livers, black arrows denote PCNA+stained cells, *n*=6 (**e**) Graph shows NIMP-R14 cells in liver sections from 40-week DEN-injured WT and *nfkb1*^*S340A*^ mice, *n*=6. Representative photomicrographs at × 200 magnification of NIMP-R14 staining in WT and *nfkb1*^*S340A*^ 40-week DEN-injured livers, black arrows denote NIMP-R14 stained cells. Data are means±s.e.m.; all scale bars, 100 μm. Statistical significance was determined using an unpaired *t*-test, *P<0.05 or ***P<0.001 compared with control.

We next generated a genetically modified mouse carrying a single alanine (Ala) mutation at Ser340 (Supplementary Fig. 6). Homozygote *nfkb1*^*S340A/S340A*^ mice were viable and developed to adulthood without obvious defects or gross pathologies. Mutant p105^S340A^ and p50^S340A^ proteins were expressed in all major organs but at reduced levels relative to WT (Supplementary Fig. 7a), although transcript levels were similar between WT and *nfkb1*^*S340A/S340A*^ liver tissues, indicating that loss of the serine residue may reduce p105/p50 translation or stability (Supplementary Fig. 7b). Of note, we did not detect significantly altered expression of other NF-κB subunits. Furthermore, although total tissue levels of p50^S340A^ were reduced, p50 and RelA DNA binding were induced to a similar degree by DEN injury at κB target sequences when comparing WT and *nfkb1*^*S340A/S340A*^ (Supplementary Fig. 7c). To assess the impact of the mutation on neutrophil recruitment, adult *nfkb1*^*S340A/S340A*^ mice were injured acutely with DEN. Highly augmented acute neutrophil inflammation was observed in the *nfkb1*^*S340A/S340A*^ background relative to WT controls (Fig. 4a). Furthermore, hepatic CXCL1, CXCL2 and S100A9 were overexpressed when compared with WT (Fig. 4b and Supplementary Fig. 7d). To determine the susceptibility of *nfkb1*^*S340A/S340A*^ mice to DEN-induced HCC, a 40-week disease model was carried out alongside WT controls. *nfkb1*^*S340A/S340A*^ males developed significantly more tumours than WT (Fig. 4c). This accelerated disease phenotype was confirmed by increased liver weight, elevated numbers of PCNA+ proliferating hepatocytes (Fig. 4c,d) and substantially higher numbers of hepatic neutrophils (Fig. 4e). We have therefore identified a single amino acid (Ser342 human or Ser340 mouse) that on mutation enhances injury-induced expression of neutrophil chemokines and increases susceptibility to HCC.

### Neutrophils promote hepatocellular telomere damage

**Figure 5:**
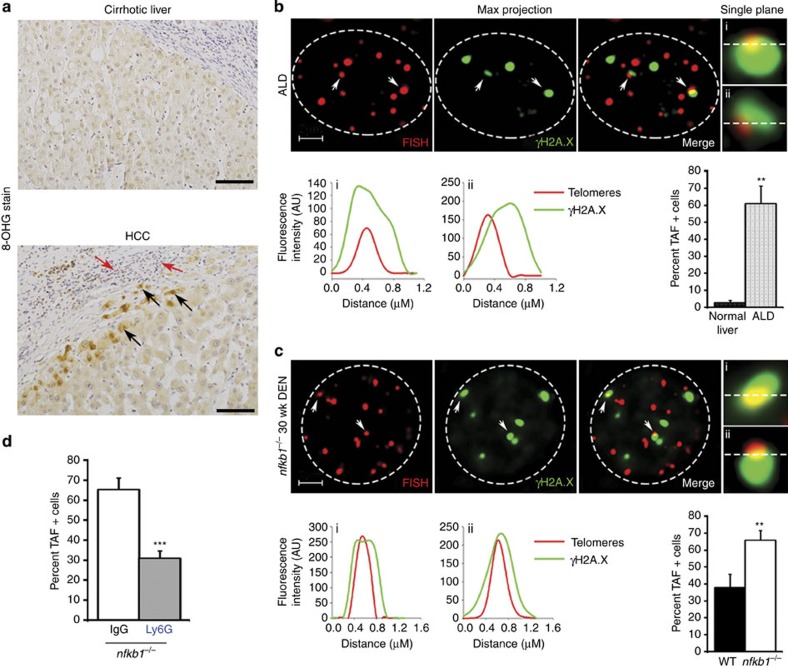
Neutrophils promote hepatocellular telomere DNA damage. (**a**) Representative photomicrographs at × 200 magnification of 8-OHG staining in liver sections from cirrhotic livers and HCC, black arrows denote brown positively stained areas of damage in hepatocytes and red arrows show inflammatory cells. Scale bars, 100 μm . (**b**) Representative deconvolved maximum intensity projections of telomere FISH and phospho-H2A.X (γH2A.X) staining in alcoholic liver disease (ALD) liver sections (*n*=2 normal human liver and *n*=4 ALD). Graph shows per cent TAF+ hepatocytes in ALD liver compared with normal control liver. (**b**,**c**) Graphs (i–ii) show the fluorescence intensity and co-localization of telomere FISH and γH2A.X staining of the corresponding single plane images. Scale bars, 3 μm . (**c**) Representative immuno-FISH images (deconvolved maximum intensity projections) from 30-week DEN-injured *nfkb1^−/−^* livers, *n*=5. Graph per cent TAF TAF+ hepatocytes in WT versus *nfkb1^−/−^* 30-week DEN livers. (**d**) Graph shows per cent TAF+ hepatocytes in 30-week DEN-injured *nfkb1^−/−^* mice treated with control IgG or Ly6G antibody for 8 weeks, *n*=10. All data are means±s.e.m. TAF analysis, a minimum of 50 cells per liver counted for human sections and a minimum of 80 cells counted per mouse section). Statistical significance was determined using an unpaired *t*-test, **P<0.01 or ***P<0.001 compared with control.

A remaining question was the nature of the neutrophil-mediated pro-tumour mechanism that is opposed by hepatocellular *nfkb1*. This is likely to be multifactorial given the vast array of cytotoxic molecules, inflammatory mediators and mitogenic factors released by neutrophils^37^. However, when examining human HCC tissue we were intrigued by the juxtaposition of neutrophil-rich inflammatory foci with hepatocytes positive for 8-hydroxyguanosine, the latter being a biomarker for oxidative DNA damage (Fig. 5a). In addition, immuno-fluorescent *in situ* hybridization (FISH) using antibody against DNA damage marker γH2A.X and FISH for the telomere-specific (C3TA2)3 PNA probe revealed the presence of tight clusters of hepatocytes with telomere-associated DNA damage in human alcoholic liver disease (Fig. 5b and Supplementary Fig. 8a). By contrast, we detected negligible telomere-associated foci (TAF+) hepatocytes in normal human liver (Fig. 5b). DNA damage in telomeres is slow to repair^38^,^39^ and these lesions contribute to loss of telomere integrity, chromosome instability and cancer^40^,^41^. This latter concept was examined experimentally in the context of HCC by Begus-Nahrmann *et al.*^42^ who reported that transient telomere dysfunction in the liver is sufficient alone to promote chromosome instability and hepatocarcinogenesis. We therefore employed immuno-FISH to examine telomere DNA damage in DEN-injured mouse livers. A higher percentage of TAF+ hepatocytes were found at 30 weeks in *nfkb1^−/−^* livers compared with WT (Fig. 5c). Comparison of TAF between WT and *nfkb1^−/−^* livers at early time points revealed no differences in numbers of TAF+ hepatocytes at 5 weeks post-DEN injury (Supplementary Fig. 8b); however, elevated numbers of TAF+ cells were detected in *nfkb1^−/−^* livers before tumour growth at 15 and 20 weeks post DEN (Supplementary Fig. 8c). Furthermore, higher numbers of TAF+ cells were found in the livers of 3-month-old *nfkb1^−/−^* mice compared with age-matched WT mice, again indicating TAFs precede the development of HCC (Supplementary Fig. 8d). Notably, *in vivo* treatment of DEN-injured *nfkb1^−/−^* mice with anti-Ly6G resulted in a significant reduction in TAF+ hepatocytes (Fig. 5d) coincident with reduced tumour burden (Fig. 1f). From these data we conclude that the aggressive inflammatory reaction that develops between 5 and 15 weeks post DEN in *nfkb1^−/−^* liver is accompanied by enhanced levels of hepatocellular telomere DNA damage, and that the neutrophilic component of the inflammatory reaction is the cellular mediator of these telomere lesions.

**Figure 6:**
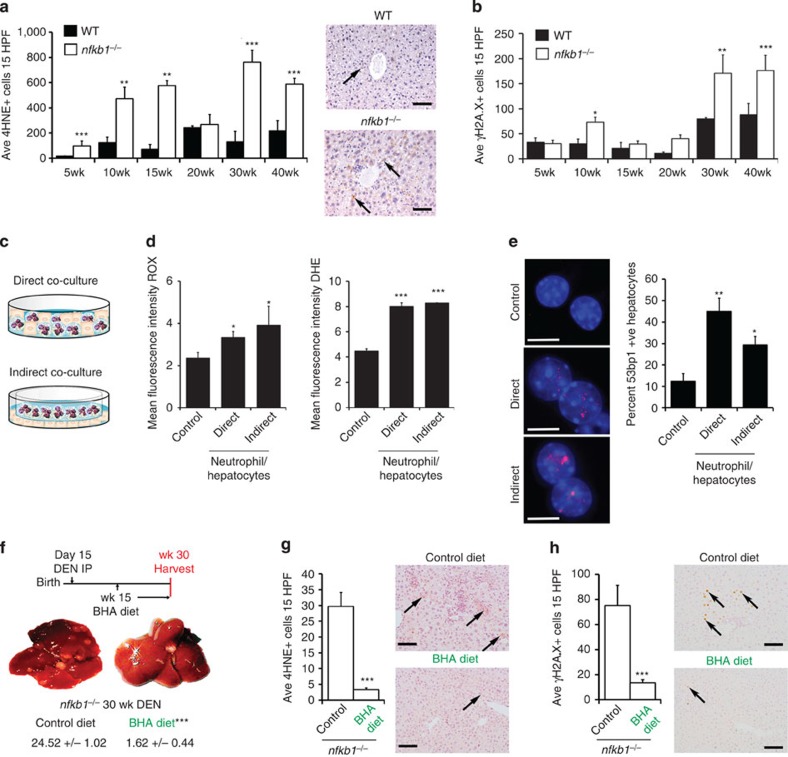
Neutrophil-dependent bystander ROS and hepatocellular cancer is limited by anti-oxidants. (**a**,**b**) Graph shows average total number of HNE- (**a**) and γH2A.X- (**b**) positive cells in liver sections from WT or *nfkb1^−/−^* mice 5–40 weeks post DEN injury (*n*=6, 4, 6, 7, 15, 9 (*nfkb1^−/−^*) and 4, 4, 4, 5, 11, 14 (WT) for the 5- to 40-week time points, respectively). (**a**) Representative photomicrographs at × 200 magnification show hepatic 4HNE staining in 40-week DEN-injured WT or *nfkb1^−/−^* mice, black arrows denote 4HNE+ hepatocytes; scale bars, 100 μm. (**c**) Diagram showing either direct (top) or indirect (bottom) transwell co-culture of WT hepatocytes and WT neutrophils. (**d**) Graphs show mean fluorescence intensity of ROX and dihydroehidium (DHE; ROS production) in WT hepatocytes cultured±WT neutrophils *n*=3. (**e**) Representative immunocytochemistry images and cell counts of DAPI/53BP1-stained WT hepatocytes only or WT hepatocytes in either direct or indirect (transwell) co-culture with WT neutrophils *n*=3; scale bars, 10 μm. (**f**) Diagram of BHA therapy in 30-week DEN-injured *nfkb1^−/−^* mice. Representative liver pictures and average tumour counts in 30-week DEN-injured *nfkb1^−/−^* mice±15 weeks dietary supplementation with BHA (*n*=5 control diet and 8 BHA diet). (**g**,**h**) Graphs show average HNE (**g**) and γH2A.X (**h**) counts and representative photomicrographs of liver sections from 30-week DEN-injured *nfkb1^−/−^* mice±BHA. Black arrows denote 4HNE+ hepatocytes (**g**) or γH2A.X+ hepatocytes (**h**); scale bars, 100 μm. All data are means±s.e.m. Statistical significance was determined using an unpaired *t*-test, *P<0.05, **P<0.01 or ***P<0.001 compared with control.

Neutrophils induce oxidative DNA damage in bystander cells by intercellular transfer of ROS^43^. Intracellular ROS causes lipid peroxidation and generation of *trans*-4-hydroxy-2-nonenol (4HNE), which following conversion to 2,3-epoxy-4HNE reacts with deoxyadenosine and deoxycytidine resulting in DNA lesions^44^. 4HNE-positive hepatocytes were consistently found in greater numbers in *nfkb1^−/−^* livers compared with WT across the 5- to 40-week DEN time course (Fig. 6a). This correlated at most time points with higher levels of hepatocellular DNA damage (γH2A.X+ cells) (Fig. 6b). *In vitro* experiments were designed to test our hypothesis that neutrophils induce bystander ROS and DNA damage in hepatocytes. Primary hepatocytes were co-cultured either in direct contact with neutrophils or were exposed to neutrophil-derived diffusible factors by means of transwell culture (Fig. 6c). Measurement of intracellular ROS within co-cultured hepatocytes revealed that either direct or indirect contact with neutrophils was sufficient to elevate hepatocellular ROS (Fig. 6d). Representative 4,6-diamidino-2-phenylindole (DAPI)/53BP1-stained hepatocytes from these co-cultures were used to quantify the percentage of hepatocytes with DNA damage foci. 53BP1+ foci were more abundant in co-cultures compared with monoculture controls (Fig. 6e and Supplementary Fig. 9a). As *in vivo* confirmation that neutrophils are contributors to ROS-induced lipid peroxidation, treatment with anti-Ly6G reduced the numbers of 4HNE+ hepatocytes (Supplementary Fig. 9b). Further *in vivo* support for ROS as a mediator of neutrophil-stimulated HCC was demonstrated by therapeutic application of the dietary anti-oxidant butylated hydroxyanisole (BHA), which protected against progression of the aggressive DEN-induced HCC disease in the *nfkb1^−/−^* background (Fig. 6f). This protective effect of BHA was accompanied by reduced 4HNE+ (Fig. 6g) and γH2A.X-stained (Fig. 6h) hepatocytes, providing a clear *in vivo* correlation between neutrophil-derived ROS, oxidative DNA damage and development of HCC.

## Discussion

Neutrophil infiltration is associated with poor prognosis in a variety of human cancers including colorectal carcinoma, head and neck squamous carcinoma, bronchioloalveolar carcinoma and HCC^45^. Here we have described how inflammatory signals released from hepatocytes in the damaged liver orchestrate the recruitment of circulatory neutrophils into the hepatic parenchyma where they stimulate genotoxic damage in bystander hepatocytes inclusive of telomere lesions that are sufficient to stimulate HCC^40^,^42^. This contrasts with an apparent lack of contribution of neutrophils to regenerative or fibrogenic responses in the injured liver^7^,^8^ and indicates that the presence of neutrophils in human ASH and NASH liver may be a risk factor for progression to HCC, making these cells potential therapeutic targets. Significant challenges will be to confirm the role of neutrophils in human CLD and to then translate this knowledge to strategies for manipulating neutrophils in the diseased liver. For the latter, opportunities include in-development small molecular inhibitors that block activities of the IL-8 family chemokines or their neutrophil receptor CXCR2 (ref. 46). Alternatively, the tumour suppressor activities of the *NFκB1* gene may be exploitable. We have presented data in support of an anti-tumour mechanism for *NFκB1* via p50:p50-mediated transcriptional repression of neutrophil-recruiting chemokines. From our observations, we predict that enhancing the anti-inflammatory properties of p50:p50 and its co-repressor HDAC1 in hepatocytes would have suppressive effects on the hepatic expression of multiple neutrophil chemokines, thereby limiting hepatic neutrophil accumulation and reducing the incidence of genotoxic and telomeric damage in bystander hepatocytes. Possible approaches would be to exploit the ability of co-factor Bcl3 to enhance stability of p50:p50 dimers at promoters^34^ or to manipulate the assembly of p50 subunits into homodimers over heterodimers. Towards the latter aim, we have begun to investigate structural features of p50 that dictate the choice of dimer partner and have identified human S342/mouse S340 as non-essential for heterodimer assembly but essential for homodimers. A mouse carrying a mutation at S340 displayed similar disease phenotypes to the *nfkb1^−/−^* mouse including increased susceptibility to HCC. We have therefore demonstrated that p50 contains discrete structural components that can be manipulated to modify its selection of dimer partner and which have an impact on inflammation-associated cancer. Therefore, more detailed structure–function studies on p50 are warranted with the aim of discovering how to experimentally promote the assembly and stabilization of anti-inflammatory p50:p50 dimers.

A caveat with the concept of inhibiting hepatic neutrophil recruitment in CLD is the potential dual role for neutrophils in cancer and the risk of removing neutrophil-mediated cytotoxic activities directed against tumour cells^47^. Disarming the harmful bystander effects of neutrophils on hepatocytes while retaining their anti-tumorigenic activities may be an optimal approach. Our discovery that neutrophils induce ROS-mediated telomere damage on bystander hepatocytes is therefore significant given the associations in the literature between telomere lesions, telomerase reactivation and cancer^42^. Telomere DNA damage foci were detected *in vivo* in diseased livers and found to be exacerbated in *nfkb1^−/−^* mice, but were ameliorated in animals treated with anti-Ly6G. *In vitro* co-culture experiments confirmed the ability of neutrophils to induce telomere DNA lesions in hepatocytes and indicated a diffusible mediator is responsible for this bystander damage. In this regard, long-lived neutrophil-derived ROS species (for example, HOCl and *N*-chloramines) and/or proteolytic enzymes are worthy of future consideration and, in particular, as they are also naturally occurring DNA repair inhibitors^48^.

It is pertinent to consider the relevance of our findings for inflammation-driven cancer in humans. The degree to which currently available rodent models of liver disease recapitulate the pathologies underpinning the development of HCC in humans is questionable. It is worth noting the absence of the ageing process in these models, which is intimately linked with CLD and HCC in humans. Ageing is associated with a physiological increase in hepatic lipid accumulation, elevated systemic expression of inflammatory mediators, loss of replicative potential for hepatocytes and a substantially increased risk for the development of fibrosis and HCC^49^. We have recently reported that the *nfkb1^−/−^* mouse has a reduced lifespan that is associated with increased levels of cellular senescence in regenerative organs including the liver, which can be ameliorated by treatment with the anti-inflammatory drug ibuprofen^22^. Here we now document in these mice the spontaneous age-dependent development of numerous lesions that are reminiscent of those found in age-associated human liver disease including steatosis, hepatitis, fibrosis, ductular proliferation, dysplasia and HCC. Further detailed investigations of the interplay between mechanisms of accelerated liver ageing and cancer in the *nfkb1^−/−^* background may be highly illuminating. Finally, although there is no obvious human model for loss of p50 homodimers, there are common polymorphisms in the human *NFKB1* gene that have been genetically linked with inflammatory disease. In particular, the –94 del/ins (rs28362491)-promoted polymorphism, which is predicted to result in reduced expression of p50, is associated with increased risk for HCC and for other cancers including breast, prostate, gastric, colorectal and oral^50^,^51^,^52^,^53^. However, as yet there are no investigative studies in the literature examining the impact of the –94 del/ins polymorphism on dimer assembly and occupancy at NF-κB-regulated genes.

## Methods

### Mice and models of liver injury

All experiments on male C57BL/6 (WT), *nfkb1*^*^−/−^*^, *s100a9*^*^−/−^*^ and *nfkb1*^S340A/S340A^ mice were performed under approval from the Newcastle Ethical Review Committee and a UK Home Office licence. *s100a9*^*^−/−^*^ mice were kindly provided by Professor Nancy Hogg (UK), *nfkb1*^*^−/−^*^ mice were a gift from Professor J. Caamano (UK). *nfkb1*^*^−/−^*^ and *s100a9*^*^−/−^*^ mice were bred in-house as homozygous lines and compared with in-house C57BL/6 mice. *nfkb1*^S340A/S340A^ homozygous knock-in mice and WT controls were derived from *nfkb1*^S340A/WT^ heterozygous knock-in mice custom made at Taconic. Day 15 mice were given 30 mg kg^−1^ N-Nitrosodiethylamine (DEN) in 0.9% saline by intraperitoneal (i.p.) injection to induce liver cancer. Acute DEN, 8-week-old mice were given 100 mg kg^−1^ by i.p. injection to induce liver DNA damage. Either power calculations or previous studies, which achieved statistical significance, were used to determine all group sizes.

### Genotyping of *nfkb1* ^S340A/S340A^ mice

Genotyping was performed by PCR using genomic DNA isolated from ear clips. Tissue sample was the digested tissue in a buffer of 0.5% SDS, 20 mM EDTA, 200 mM NaCl, 40 mM Tris pH8.0, with 50 μg ml^−1^ proteinase K at 55 °C overnight, followed by phenol/chloroform extraction and DNA precipitation. The WT or *nfkb1*^S340A/S340A^ alleles were amplified using NeoDel forward 5′-GTCTTCAAAACGCCAAAGTATAAGGATGTC-3′ and NeoDel reverse 5′-CCCCTCCTGGTGGAGGACCAC-3′ specific primers for 30 cycles; denaturation: 98 °C, 10 s; annealing: 58 °C, 15 s; extension: 68 °C, 1 min). Amplification of a single product of 490 or 620 bp corresponds to WT or 340^+/+^ allele, respectively. Amplification of both products corresponds to a heterozygous (+/−) genotype (Supplementary Fig. 6a–c).

### Therapies

Mini-pumps (Alzet, model 2004) were implanted subcutaneously into 22-week DEN-injured (*nfkb1*^*^−/−^*^) or 32-week DEN-injured (WT) mice to deliver 28.5 μg per day of Ly6G (clone 1A8) neutrophil depleting antibody or Rat IgG2a (clone 2A3) (BioXCell) for 8 weeks. *nfkb1*^*^−/−^*^ mice were fed BHA (0.7% w/w) or normal chow 15 weeks post DEN for 15 weeks. CXCL1 and CXCL2 were neutralized by administering i.p. 25 μg of anti-CXCL1 (AF-453-NA) and 25 μg of anti-CXCL2 (AF-452-NA) or 50 μg goat IgG (AB-108-C) 1 h before and 18 h post acute DEN. At the end of the experiment, animals were culled, and the liver and serum harvested for analysis.

### *In vivo* imaging

WT neutrophils were labelled using the Cell Vue NIR815 fluorescent kit (Li-Cor), according to manufacturers instructions, then 1 × 10^7^ cells were injected intravenously via the tail vein into 18 h acute DEN-injured WT or *nfkb1*^*^−/−^*^ mice. The IVIS220 series imaging system (Ex max 786 nm; em max 814 nm filters) was used to longitudinally track neutrophil migration; after the final scan, animals were culled and the liver, kidney and the spleen were removed and imaged *ex vivo*. Data were analysed using Living Image 4.2 software, regions of interest were drawn and Average Radiant Efficiency (p s ^−1^cm^−2^ sr^−1^)/(μW cm^−2^) was calculated.

### Neutrophil isolation

Briefly, bone marrow was extracted from the femur and tibia of WT, *nfkb1*^*^−/−^*^ and *nfkb1*^S340A/S340A^ mice by flushing with HBBS–Ca^2+^ with 5% serum. Neutrophils were isolated by percoll gradient (62%) and purity was established by Ly6G and CD11b (BD Biosciences) flow cytometry (BD FACScantoII). We used the 7AAD and Annexin V apoptosis detection kit according to manufacturers instruction (BD Biosciences) and flow cytometry to measure neutrophil apoptosis.

### Generation of bone marrow chimera

Eight- to 12-week-old WT and *nfkb1*^*^−/−^*^ mice were used as donors and recipients. Bone marrow cells were isolated from WT and *nfkb1*^*^−/−^*^ mice as described above. The bone marrow cell suspension was washed, centrifuged at 400*g* for 5 min, then re-suspended and counted. Recipient mice underwent whole body irradiation (NDT 320KV 3.2KW irradiator) at 10 Gy and then received 10^7^ bone marrow cells intravenously.

### Hepatocyte isolation

Hepatocytes were isolated from the livers of WT, *nfkb1*^*^−/−^*^ and *nfkb1*^S340A/S340A^ mice by digestion with collagenase from *Clostridium histolyticum* (Sigma), then filtered through a 70-μm cell strainer. Cells were collected by centrifugation (500 r.p.m. for 3 min), washed three times in Krebs–Ringer buffer (Sigma) and then re-suspended in Williams medium E with 10% serum (WME Gibco), and plated onto collagen-coated plates (type I collagen, BD Biosciences). After 4 h, medium was removed and cells were cultured in fresh 10% or 0.5% Williams medium E.

### Hepatocyte and neutrophil co-culture and ROS production

WT and *nfkb1*^*^−/−^*^ hepatocytes were plated on collagen-coated transwell plates, then co-cultured with WT or *nfkb1*^*^−/−^*^ neutrophils±a 3-μm transwell insert (Thincert Griener) at a 1:1 ratio for 18 h. Intracellular ROS of hepatocytes and isolated neutrophils was measured following incubation with 10 μM difluorofluorescein diacetate (FITC 488 nm), 5 μM dihydroehidium (Rhodamine, 594 nm) or 5 μM Cell Rox Orange for 30 min at 37 °C. Median fluorescence intensity was measured by flow cytometry. Up to 10,000 events were analysed on FACScan/FACS Canto II (BD, Oxford, UK) using Flowjo software (FlowJo, Inc.).

### Bone marrow macrophages

Bone marrow cells from the femurs of WT or *nfkb1*^*^−/−^*^ mice were differentiated into macrophages (7 days with RPMI media supplementedwith 5% horse serum (Sigma-Aldrich) and 10% L929 supernatant (refreshed on days 3 and 6)).

### Cell culture

Cos-7 cells were cultured in DMEM supplemented with 100 U ml^−1^ penicillin, 100 μg ml^−1^ streptomycin, 2 mM L-glutamine and 16% fetal bovine serum at 37 °C at an atmosphere of 5% CO_2_.

### Adenoviral expression of WT and S340A p50

Hepatocytes were transduced with one green fluorescent forming unit per cell of control adenovirus or adenovirus expressing either WT murine p50 or S340A p50 for 24 h. Adenovirus was kindly provided by Professor G Eric Blair (UK).

### Human tissue

HCC and alcoholic liver disease liver samples for histology were collected under full ethical approval with patient consent (REC references 10/H0906/41).

### ChIP assay

Cross-linked chromatin was prepared from acute DEN-injured WT, *nfkb1*^*^−/−^*^ and *nfkb1*^S340A/S340A^ mouse livers, using the protocol outlined in the Upstate Biotechnology Immuno-precipitation ChIP assay kit. ChIP was performed using 25 μg of cross-linked chromatin per reaction and 5 μg of antibody to HDAC1 (05-100 Millipore), p50 (ab7971 Abcam) or IgG control (Abcam) for immunoprecipitation. Mouse S100A9, CXCL1 and two promoters were amplified by quantitative reverse transcriptase–PCR using specific primers (Supplementary Methods Table 1).

### Electromobility shift assay

Cos-7 cells were co-transfected with 1 μg Flag-tagged WT-p50 or mutant-p50 using the effectene kit (Qiagen). After 48 h, cells were lysed with Dignam A buffer and incubated on ice for 15 min with vortexing, then centrifuged at 900*g* at 4 °C for 30 s. The cell pellet was re-suspended in Dignam C buffer and incubated on ice for 15 min. After centrifugation at 900*g* (4 °C) for 5 min nuclear extracts were removed and stored at −80 °C. p50 For EMSA, briefly 8–10 μg of nuclear extract was incubated with poly dIdC and ^32^P-labelled NFκB Oligonucleotide (Promega) for 20 min at 4 °C. For supershift assays, reactions were incubated in the presence of 2 μg of anti-flag or anti-myc as a negative control (Sigma) for 16 h at 4 °C. EMSA and supershift reaction mixtures were resolved by electrophoresis on an 8% non-denaturing polyacrylamide gel (37:5:1).

### Enzyme-linked immunosorbent assay

WT and *nfkb1*^*^−/−^*^ hepatocytes were cultured overnight before protein extraction from the pelleted cells. Twenty micrograms of protein was used for CXCL2/MIP2 ELISA. The ELISA was performed according to the manufacturer's instructions (R&D Quantikine ELISA kit).

### Histological stains

Formalin fixed, paraffin-embedded liver sections were stained with haematoxylin and eosin and 0.1% Sirius Red Picric solution following standard procedures. Diastase PAS, Reticulin and Sirius red/fast green, keratin 19 and glutamine synthetase were immunostained at the Department of Cellular Pathology, Royal Victoria Hospital, Newcastle Upon Tyne.

### Immunohistochemistry

Staining was performed on formalin-fixed, paraffin-embedded liver sections. Endogenous peroxidase activity was blocked with hydrogen peroxide and antigen retrieval was achieved using proteinase K (20 μg ml^−1^) for detection of F480 1:100 (ab6640 Abcam), 0.01% pronase for neutrophil elastase 1:200 (ab21595 Abcam) and NIMP-R14 1:200 (ab2557 Abcam), and antigen unmasking solution for active caspase-3 1:200 (9664 Cell Signaling), S100A9 1:100 (ab73987 Abcam), 4HNE 1:50 (MHN-020P JalCA), αSMA 1:1000 (F3777 Sigma) and Collagen IV 1:100 (Abcam). EDTA (1 mM) for γH2A.X 1:100 (9718 Cell Signaling) and trypsin for PCNA 1:250 (ab2426 Abcam). Tissue was blocked using an Avidin/Biotin Blocking Kit (Vector Laboratories) followed by 20% swine serum in PBS and then incubated with primary antibodies overnight at 4 °C. The next day, slides were washed and incubated with biotinylated swine anti-rabbit 1:200 (eo353 Dako), biotinylated goat anti-fluorescein 1:300 (BA-0601 Vector) or goat anti-rat 1:200 (STAR80B Serotec), followed by Vectastain Elite ABC Reagent. Antigens were visualized using DAB peroxidase substrate kit and counterstained with Mayer's haematoxylin. For 4HNE, amplification was achieved using the mouse on mouse kit (Vector) Immuno-stained cells were manually counted and expressed as the mean number of positive cells in 15 high power ( × 20) fields. Image analysis of a minimum of ten fields ( × 10) was performed using Leica Qwin for F4/80. All stained histological sections were blinded before counting/analysis.

### Histological assessment of liver disease and cancer

Histological examination of aged WT and *nfkb1^−/−^* liver sections was performed by a liver pathologist (DGT) and immunostains were evaluated by two observers (DGT and FO). Steatosis severity, the presence of steatohepatitis and extent of fibrosis were assessed according to Kleiner *et al.*^54^ Evaluation of hepatocellular dysplasia and neoplasms was based according to established histological criteria^55^.

### Telomere immuno-FISH

Formalin-fixed paraffin-embedded liver sections were dewaxed and hydrated, washed with water and then citric saline antigen retrieval was performed. Slides were blocked with normal goat serum (1:60) in BSA/PBS and incubated with rabbit γ-H2A.X (1:250) at 4 °C overnight. The next day, slides were washed three times in PBS, incubated with secondary antibody for 30 min, washed three times in PBS and then incubated with Avidin DCS (1:500) for 20 min. After γ-H2A.X immunofluorescence, slides were washed three times in PBS, cross-linked with 4% paraformaldehyde for 20 min and dehydrated in graded ethanol. Sections were denatured for 10 min at 80 °C in hybridization buffer (70% formamide (Sigma), 25 mM MgCl_2_, 0.1 M Tris pH 7.2, 5% blocking reagent (Roche) containing 2.5 μg ml^−1^ Cy-3-labelled telomere-specific (5′-CCCTAA-3′) peptide nuclei acid probe (Panagene), followed by hybridization for 2 h at room temperature in the dark. Slides were washed twice with 70% formamide in 2 × SSC for 15 min, followed by 2 × SSC and PBS washes for 10 min. Next, sections were incubated with DAPI, mounted and imaged. In-depth *Z* stacking was used (a minimum of 40 optical slices with × 63 objective, Leica DM5500B) followed by Huygens (SVI) deconvolution. Whole-image stacks were used to count TAFs.

### Immunocytochemistry

Hepatocytes were cultured on sterile collagen-coated coverslips, then fixed in 2% paraformaldehyde for 10 min and then blocked cells for 45 min with 0.2% Fish skin Gelatine, 0.5% BSA and 0.5% Triton X-100 in PBS. Slides were then incubated with the rabbit polyclonal anti-53BP1 4 μg ml^−1^ (NB100-904 Novus Biologicals) overnight at 4 °C. The next day, the cells were washed and incubated with Alexa Fluor 594 secondary antibody (Invitrogen) for 60 min before mounting with DAPI mountant (Vector).

### RNA isolation and real-time PCR

Total RNA was isolated from mouse liver or cultured cells using the Total RNA Purification Kit (QIAGEN) and then treated with DNAse and used as a template in first-strand complementary DNA synthesis using random primers (Promega). SYBR Green quantitative reverse transcriptase–PCR was performed using the primers listed in (Supplementary Table 5).

### Generation of p50 constructs

Flag-p50 and HA-p50 was sub-cloned into pCDNA3 using HindIII and ApaI restriction enzyme digestion and used as a template to generate the following Flag- and haemagglutinin (HA)-tagged p50 mutant constructs: p50 T145A mutant, p50 S210A mutant, p50 T315A mutant, p50 S337A mutant and p50 S342A mutant by two-step site-directed mutagenesis. A common Flag tag or HA tag forward primer was used with the respective mutant reverse primer. A common reverse primer was used for the second PCR reaction. Final PCR products were restriction enzyme digested with HindIII and ApaI, separated on 1% agarose gel, excised, purified and sub-cloned them into pCDNA3. The primer sequences used are listed in (Supplementary Table 6).

### Co-transfection and immunoprecipitation

To assess p50:RelA interactions, Cos-7 cells were co-transfected with 0.5 μg RelA-eGFP and 1 μg Flag-tagged WT-p50 or mutant-p50. For p50:p50 interactions, Cos-7 cells were co-transfected with 0.5 μg of Flag-tagged WT-p50 or mutant-p50, and 1 μg of HA-tagged WT-p50 construct. The Flag immunoprecipitation kit (Sigma) was used according to the manufacturer's instructions. At 36 h post transfection, cells were PBS washed and then lysed into RIPA buffer (input control) or lysed with Flag lysis buffer (50 mM Tris HCl, pH 7.4, with 150 mM NaCl, 1 mM EDTA and 1% Triton X-100). Flag beads were washed in wash buffer (50 mM Tris HCl pH 7.4 with 150 mM NaCl) and then 1 mg of cell lysate was added to the beads. Cell lysates and beads were agitated overnight at 4 °C and then centrifuged at 13,000 r.p.m. for 30 s. The supernatant was removed and the remaining beads were washed 3 × in wash buffer. Protein was then eluted by adding 100 μl of 3 × Flag peptide and incubated at 4 °C for 30 min with gentle agitation. The beads were centrifuged and the supernatant removed. Thirty micrograms of input control and 50 μl of IP eluate was loaded onto a 9% SDS–PAGE gel for western blotting.

### Isolation of whole-tissue lysates

Tissue (∼5 mg) was lysed and homogenized in 300 μl RIPA buffer supplemented with protease and phosphatase inhibitor cocktails. Lysates were passed through a QIAshredder and then spun at 16,000*g* for 2 min. Flow through was collected and sonicated for 10 s. Debris was pelleted by centrifugation at 16,000*g* at 4 °C for 15 min and the supernatant was collected. Protein concentration was measured using the detergent-compatible protein assay kit purchased from Bio-Rad.

### SDS–polyacrylamide gel electrophoresis

Total protein was fractionated by 9% SDS–PAGE, transferred onto nitrocellulose and then blocked blots with Tris-buffered saline and Tween 20 (0.1%) containing 5% BSA before incubation overnight with primary antibodies (1:1,000) to S100A9 (ab73987 Abcam), PCNA (ab2426 Abcam), Cyclin D1 (ab16663 Abcam), p105/p50 (ab7971 Abcam), RelA (ab7970 Abcam), c-Rel (sc71 Santa Cruz), CyP2E1 (ab28146 Abcam) or GAPDH (Abcam), total p38α (9218 Cell Signaling), P-p38α (9216 Cell Signaling), HA or Flag-HRP conjugate (Sigma). Next day, membranes were washed in T-TBS and then incubated with horseradish peroxidase-conjugated mouse anti-rabbit IgG. Blots were washed and antigen was detected using enhanced chemiluminescence (Amersham Biosciences). Images have been cropped for presentation. Full-size images are presented in Supplementary Fig. 10.

### Model of p50 homodimer binding DNA

Molecular graphics images were generated using PyMOL (DeLano Scientific) and solvent-accessible surface areas calculated with Stride^56^.

### Statistical analysis

Data are expressed as means±s.e.m. GraphPad Instat was used to perform unpaired Students *t*-test or analysis of variance with a Tukey's *post-hoc* test and *P<0.05, **P<0.01 or ***P<0.001 was considered significant.

## Authors contributions

C.L.W. carried out the majority of the laboratory-based work and analyses presented in the manuscript. D.J., N.F., P.B., S.L., A.M.E., R.G.G., J.B.C., C.F., A.L., J.F.P., G.S., K.C., A.P., C.R. and J.M. performed a portion of the laboratory experiments and their related analyses. A.J.M. was responsible for *in silico* analyses of the p50 protein. G.E.B. and N.F. produced the p50-expressing adenoviruses. F.O., J.M. and C.L.W. carried out all of the *in vivo* experiments. D.T. performed the routine histological examination and scoring of livers from the ageing mice and F.O. carried out histological and statistical analyses. J.M. produced all of the final figures. F.O. and D.A.M. conceived the studies, designed the experiments, were chiefly responsible for data interpretation and wrote the manuscript. All authors read and commented on the final manuscript.

## Additional information

**How to cite this article:** Wilson C. L. *et al.* NFκB1 is a suppressor of neutrophil-driven hepatocellular carcinoma. *Nat. Commun.* 6:6818 doi: 10.1038/ncomms7818 (2015).

## Supplementary Material

Supplementary Information Supplementary Figures 1-10 and Supplementary Tables 1-6
