# Corrigendum: *NFκB1* is a suppressor of neutrophil-driven hepatocellular carcinoma

**DOI:** 10.1038/ncomms9411

**Published:** 2015-09-21

**Authors:** C. L. Wilson, D. Jurk, N. Fullard, P. Banks, A. Page, S. Luli, A. M. Elsharkawy, R. G. Gieling, J. Bagchi Chakraborty, C. Fox, C. Richardson, K. Callaghan, G. E. Blair, N. Fox, A. Lagnado, J. F. Passos, A. J. Moore, G. R. Smith, D. G. Tiniakos, J. Mann, F. Oakley, D. A. Mann

Nature Communications 6, Article number: 6818 10.1038/ncomms7818 (2015); Published 04162015; Updated 09212015

The diagram in Supplementary Fig. 6a illustrating the targeting strategy for the *nfkb1*^*S340A*^ KI allele depicts an unrelated targeting strategy. The correct version of the figure appears below.


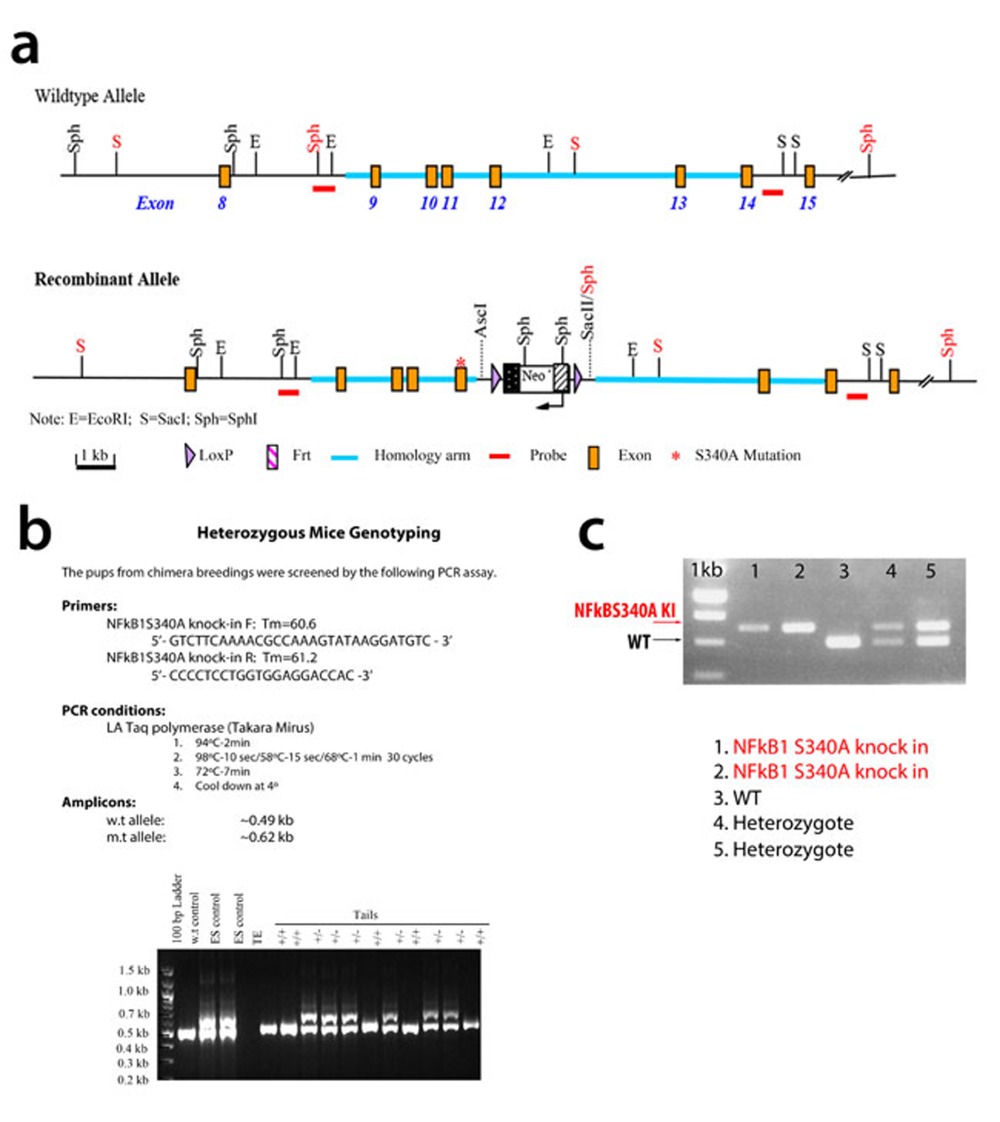


**Supplementary Figure 6**

