# Supplementary material for: NFκB1 is a suppressor of neutrophil-driven hepatocellular carcinoma
Source: Nat Commun. 2015 Apr 16;6:6818. doi: 10.1038/ncomms7818 (PMC4410629; doi:10.1038/ncomms7818)
Supplement: Supplementary Information — Supplementary Figures 1-10 and Supplementary Tables 1-6 [file ncomms7818-s1.pdf]

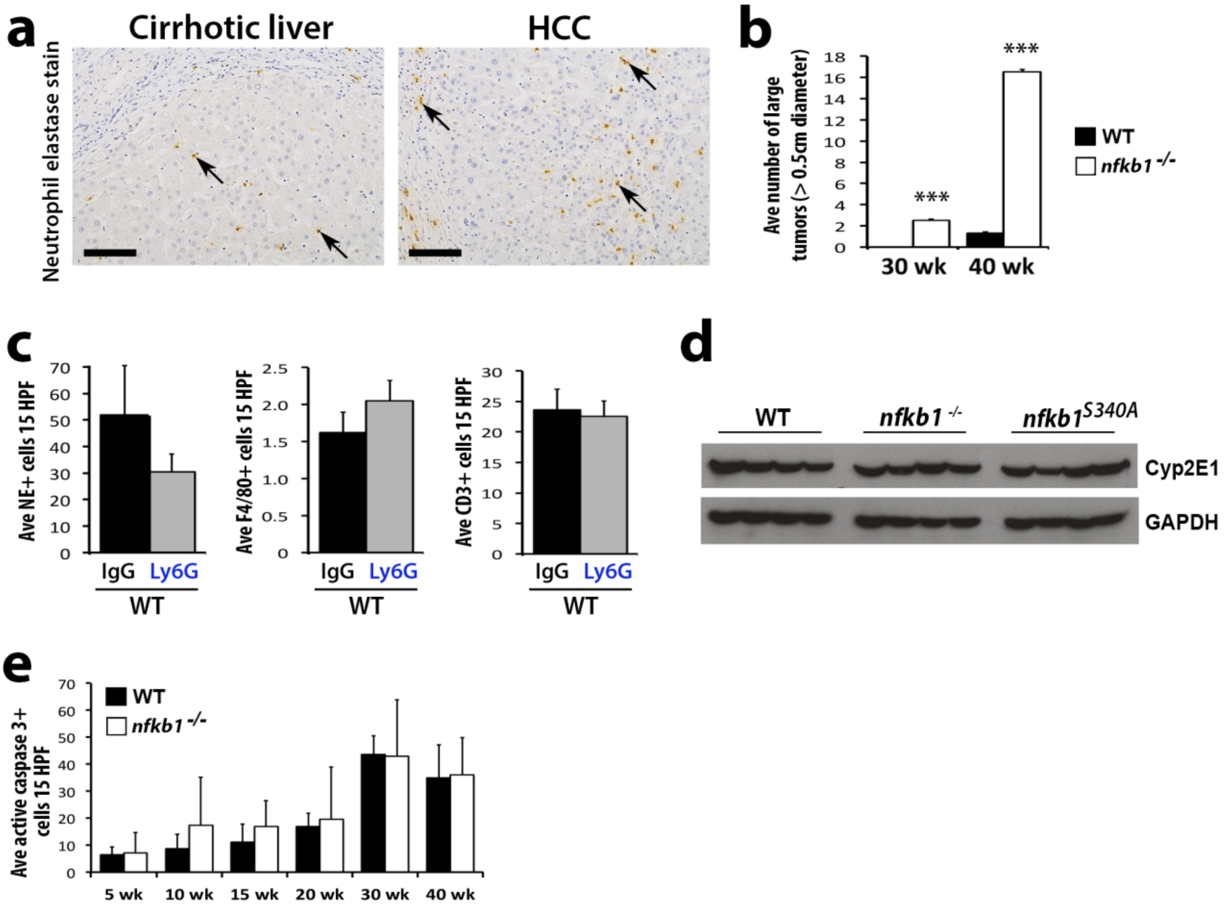

Supplementary figure 1: Neutrophils are a feature of liver cancer. (a) Representative photomicrographs at ×200 magnification of neutrophil elastase staining in liver sections from cirrhotic liver and hepatocellular carcinoma (HCC), black arrows denote brown positively stained neutrophils, scale bars are 100 microns. (b) Graph shows average number of large tumors (>0.5cm) in livers of 30 and 40 wk DEN injured WT and *nfkb1-/-* mice. (c) Graphs show the average total number of neutrophils (NE), macrophages (F4/80) and T-lymphocytes (CD3) in liver sections from WT DEN injured mice treated ± control IgG or Ly6G. (d) Western blots showing hepatic Cyp2E1 and GAPDH (loading control) levels at 24 h post DEN injury in WT, *nfkb1-/-* and *nfkb1*^{S340A}/^{S340A} mice, n=4. (e) Graph shows average total number of active Caspase 3+ hepatocytes in liver sections from WT or *nfkb1-/-* mice 5-40 wks post DEN injury. All data are means ± s.e.m. For chronic DEN experiments n=6, 4, 6, 7, 15, 9 (*nfkb1-/-*) 4, 4, 4, 5, 11, 14 (WT) for the 5–40 wk time points respectively. Statistical

significance was determined using an unpaired t-test, $^{**}P < 0.01$ or $^{***}P < 0.01$ compared to control.

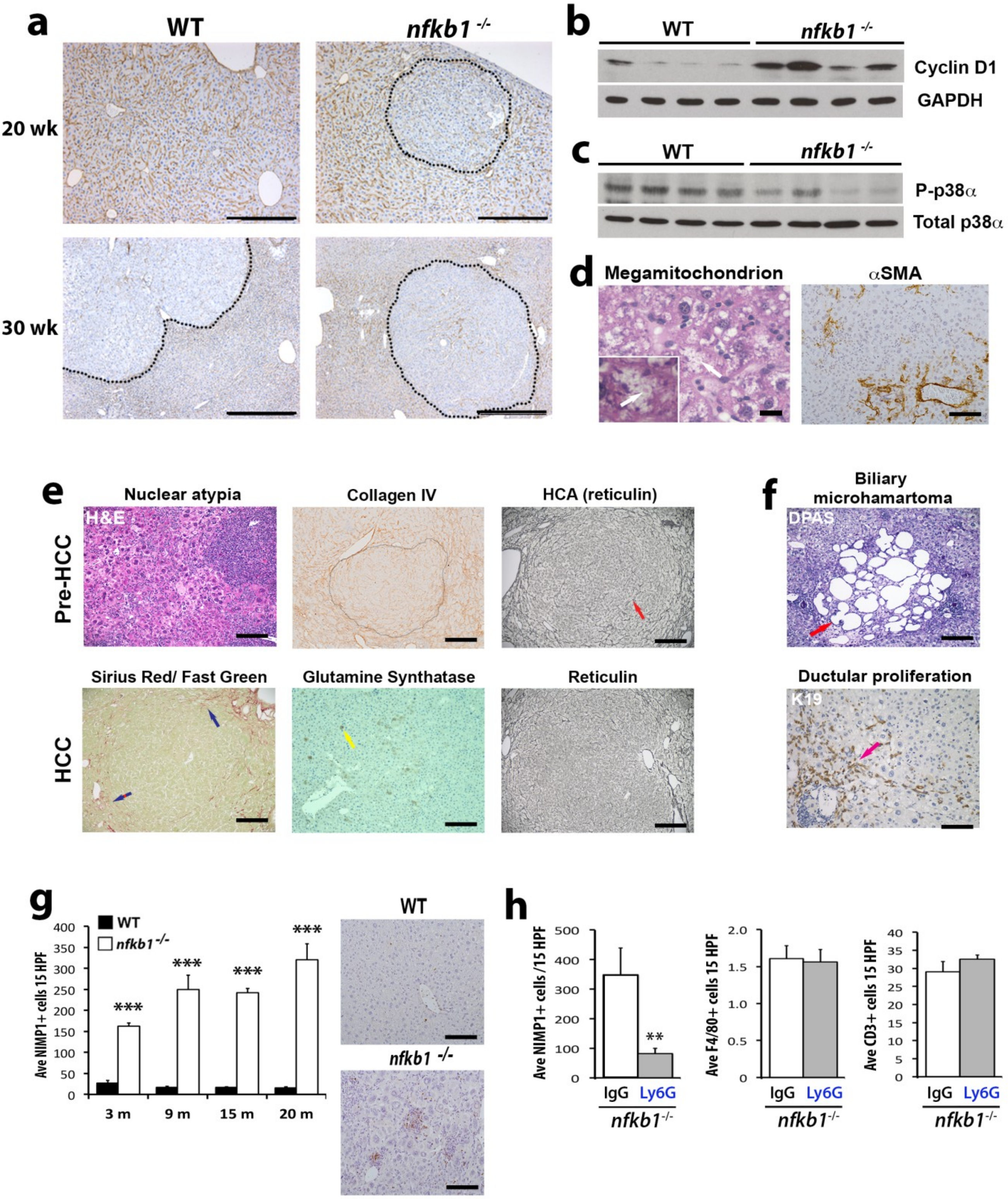

Supplementary figure 2: Characterising HCC development in DEN treated or aged WT and *nfkb1-/-* mice.

(a) Representative photomicrographs at ×100 magnification of collagen IV stained livers from 20 and 30 wk DEN injured WT and *nfkb1-/-* mice, dotted lines denote the collagen IV negative tumor areas, scale bars are 200 microns. (b-c) Western blots for Cyclin D1 and GAPDH (b) and P-p38α and total p38α (c) in whole liver lysates from 30 wk DEN injured WT and *nfkb1-/-* mice, n=4. (d) Representative photomicrographs at ×100 magnification of liver sections from 20 month aged WT and *nfkb1-/-* mice stained with H+E showing megamitochondrion (white arrows) and αSMA (hepatic myofibroblasts). (e) 20 month aged *nfkb1-/-* mice; upper panel; representative photomicrographs show inflammation and nuclear atypia (H+E), loss of collagen IV (Col IV) staining and a hepatocellular adenoma (HCA, red arrow). Lower panel; in 4 out of 5 mice with tumors develop portal/periportal and/or bridging fibrosis (Sirius red/fast green, blue arrows). HCC was associated mild diffuse glutamine synthetase immunostaining (yellow arrow) and loss of reticulin staining. (f) Images of Periodic acid–Schiff–diastase (DPAS) and Keratin 19 (k19) stained livers from aged *nfkb1-/-* show the formation of a biliary microhamartoma (red arrow) in 2 cases and extensive ductular proliferation (pink arrow). (g) Graph shows average total number of neutrophils (NIMP1+) in liver sections from 3, 9, 15 and 20 month aged WT and *nfkb1-/-* mice, and representative images of NIMP1+ stained cells in 20 month aged WT and *nfkb1-/-* livers, n= 5-8 WT and 5-10 *nfkb1-/-* mice. (h) Graphs show the average total number of neutrophils (NE), macrophages (F4/80) and T-lymphocytes (CD3) in liver sections from *nfkb1-/-* DEN injured mice treated ± control IgG or Ly6G. All data are means ± s.e.m, scale bars (d-g) are 100 microns. Statistical significance was determined using an unpaired t-test, *$P < 0.05$ or **$P < 0.01$ compared to WT control.

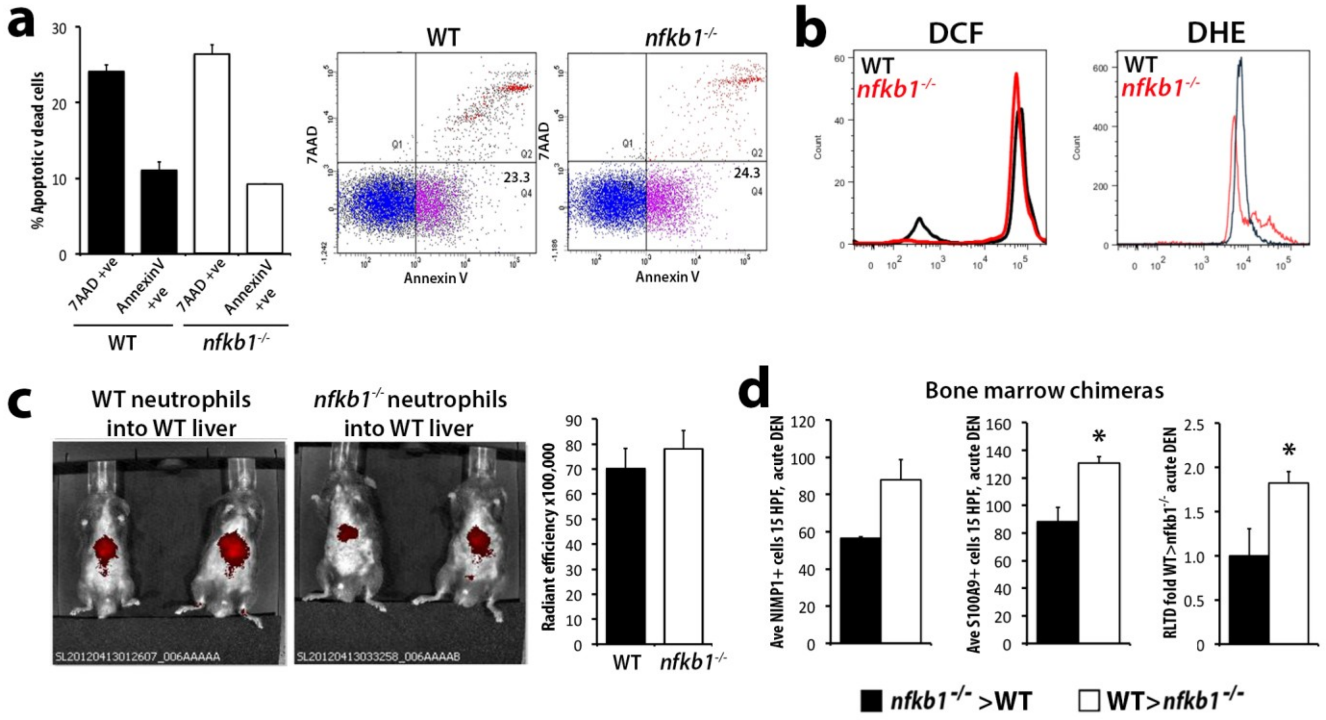

Supplementary figure 3: Neutrophil survival, ROS production and tracking are similar between WT and *nfkb1-/-* mice.

(a) Graph shows average percentage of apoptotic WT and *nfkb1-/-* neutrophils. Apoptosis was quantified by FACS using 7AAD and Annexin V staining. (b) Mean fluorescence index of DCF and DHE staining in WT and *nfkb1-/-* neutrophils was quantified by FACS. (c) Representative IVIS pictures of mice given NIR815 labeled WT or *nfkb1-/-* neutrophils I.V. showing neutrophils tracking to the liver of acute DEN injured WT mice. Graph shows average radians from IVIS imaged WT mice receiving WT or *nfkb1-/-* neutrophils. (d) Graphs show average total number of NIMP1+ and S100A9+ stained cells and S100A9 mRNA levels in livers from bone marrow chimeric mice; either WT mice receiving *nfkb1-/-* bone marrow (nfkb1>WT) or *nfkb1-/-* mice receiving WT bone marrow (WT> nfkb1) after 48h acute DEN injury, n=3 WT and 3 *nfkb1-/-* recipient mice. All data are means ± s.e.m. and representative of five mice per group.

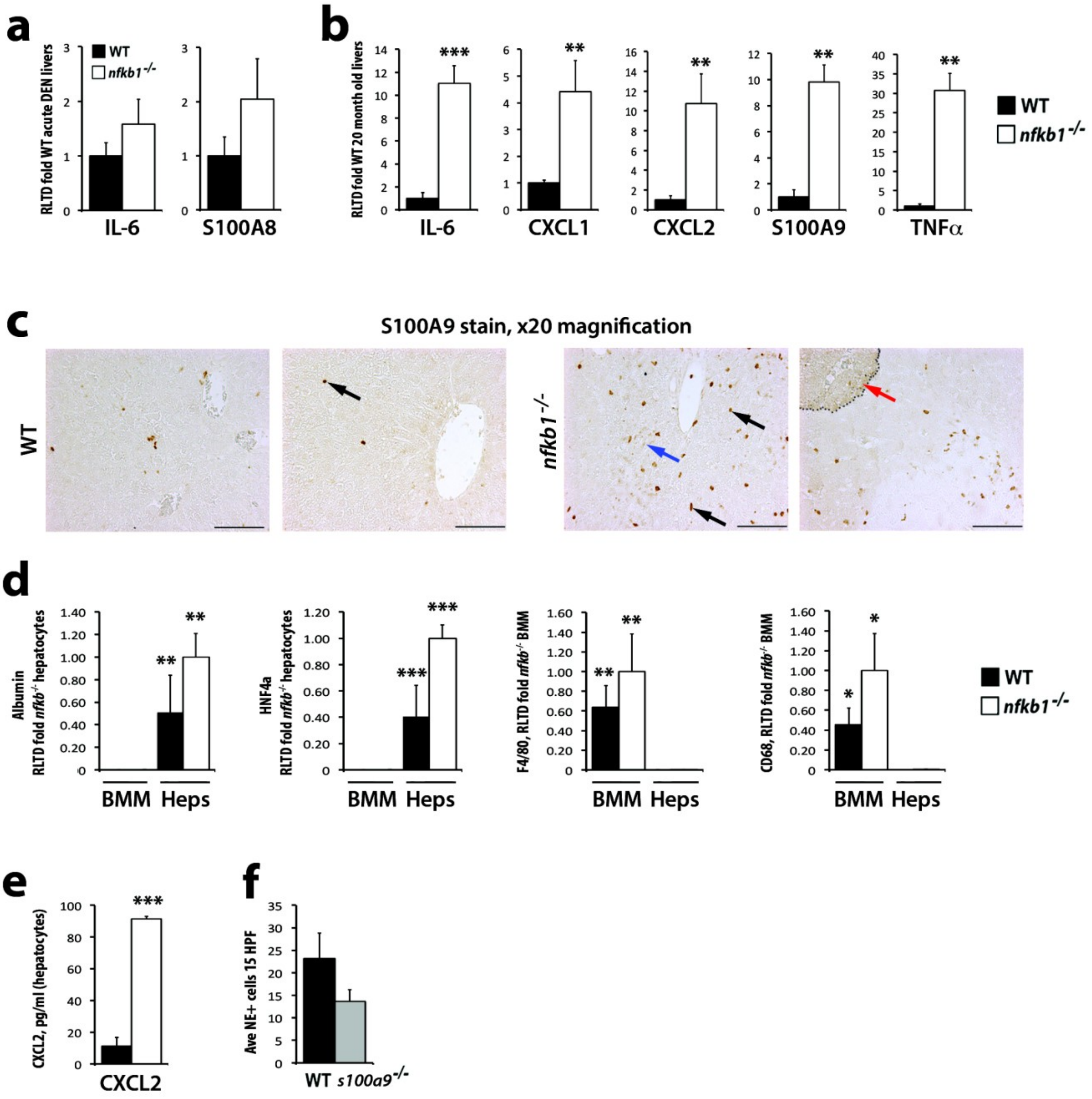

Supplementary figure 4: Deletion of *nfkb1* causes elevation of hepatic cytokines and chemokines.

(a) Hepatic IL-6 and S100A8 mRNA levels expressed as RLTD compared to WT, 48 hrs after acute DEN injury in WT and *nfkb1-/-*mice, n=6. (b) Hepatic IL-6, CXCL1, CXCL2, S100A9 and TNFα mRNA levels expressed as RLTD compared to WT in 20 month WT and *nfkb1-/-*mice, n=10. (c) Representative photomicrographs at ×100 magnification of S100A9 stained livers from 40 wk DEN injured WT and *nfkb1-/-* mice, dotted line denotes an S100A9+ tumor in the

nfkb1-/- mice. Black arrows show S100A9+ neutrophils, red arrows denote S100A9+ stained tumor and blue arrows highlight S100A9+ hepatocytes. Scale bars are 100 microns. (d) Graphs show RLTD of albumin, HNF4α, F4/80 and Cd68 in bone marrow macrophages (BMM) compared to hepatocytes isolated from WT and *nfkb1-/-* mice, n=3. (e) CXCL2 ELISA performed on hepatocytes isolated from WT and *nfkb1-/-* mice n=3. (f) Graph shows average total number of neutrophil elastase+ cells in liver sections from WT or *s100a9-/-* mice after acute DEN injury, n=12 WT and 5 *s100a9-/-* mice. All data are means ± s.e.m. Statistical significance was determined using an unpaired t-test, *$P < 0.05$, **$P < 0.01$ or ***$P < 0.001$ compared to WT control.

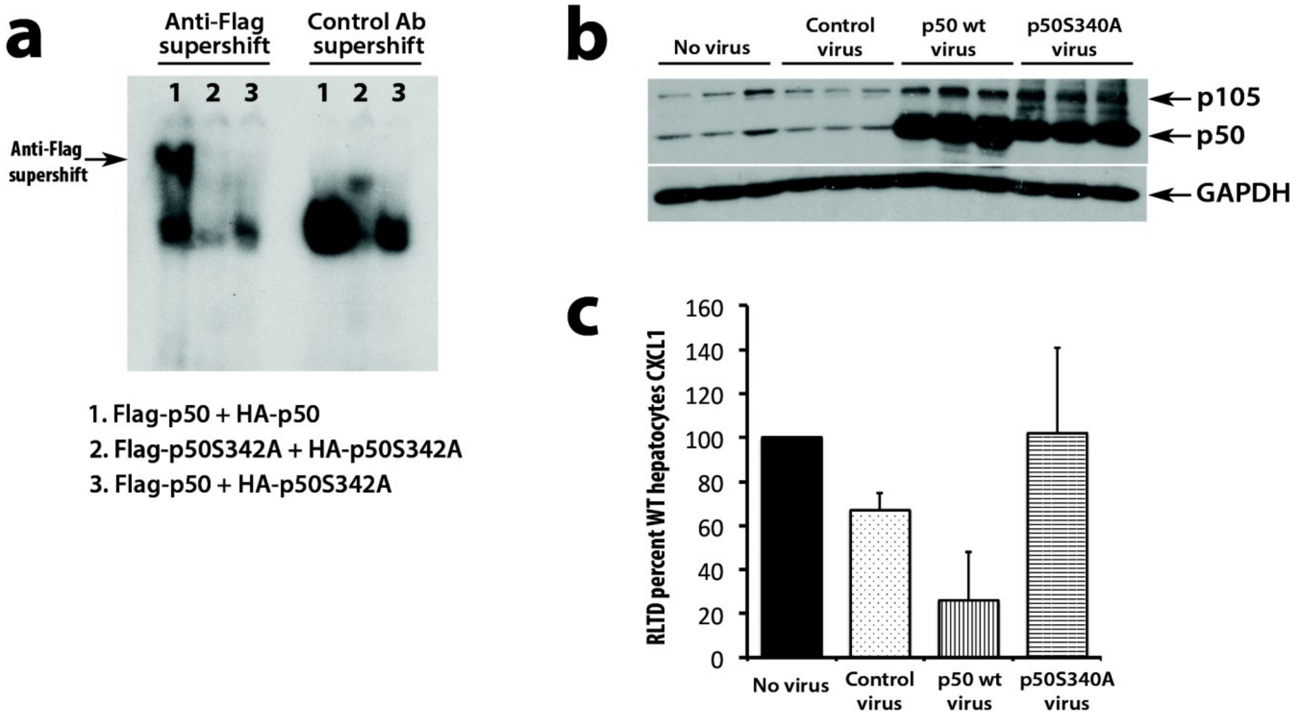

Supplementary figure 5: Mutation of nfkb1 serine 340 alanine generates a p50 that cannot form homodimers. (a) NF-κB Electromobility Shift Assay (EMSA) and Flag Supershift on nuclear extracts isolated from Cos-7 cells transfected with Flag or HA tagged human p50 or p50S342A mutant. Data is representative of at least two independent transfections. (b) Western blot showing p105/p50 levels in hepatocytes only or transduced with a control, murine p50 or murine p50S340A mutant adenovirus, GAPDH is the loading control. (c) CXCL1 mRNA levels in hepatocytes transduced with control, murine p50 or murine p50S340A mutant adenovirus for 24 hrs, data are means ± s.e.m generated from three separate hepatocyte isolations.

a

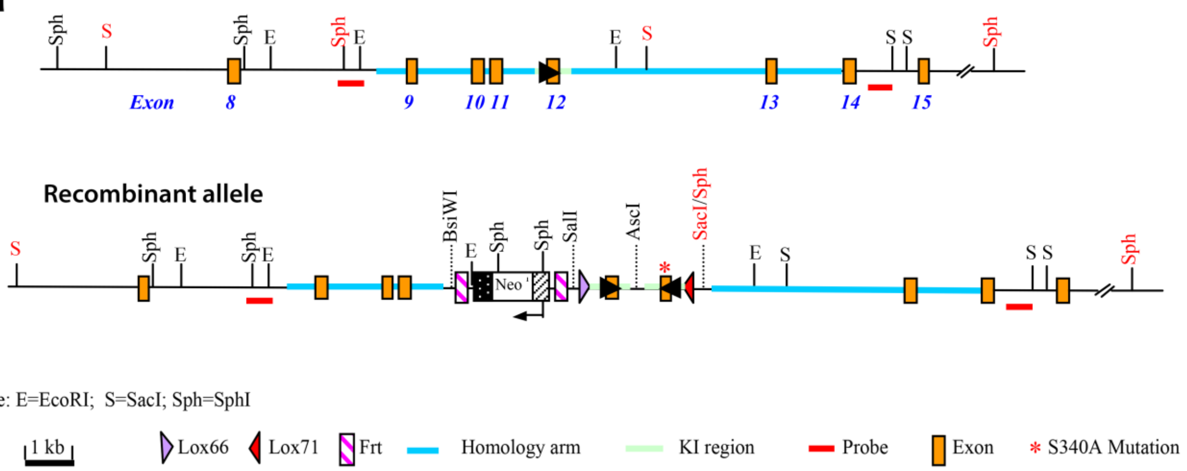

b

Heterozygous Mice Genotyping

The pups from chimera breedings were screened by the following PCR assay.

Primers:

NFkB1S340A knock-in F: Tm=60.6
5'- GTCTTCAAAACGCCAAAGTATAAGGATGTC - 3'

NFkB1S340A knock-in R: Tm=61.2
5'- CCCCTCCTGGTGGAGGACCAC -3'

PCR conditions:

LA Taq polymerase (Takara Mirus)

1. 94°C-2min
2. 98°C-10 sec/58°C-15 sec/68°C-1 min 30 cycles
3. 72°C-7min
4. Cool down at 4°

Amplicons:

w.t allele: ~0.49 kb
m.t allele: ~0.62 kb

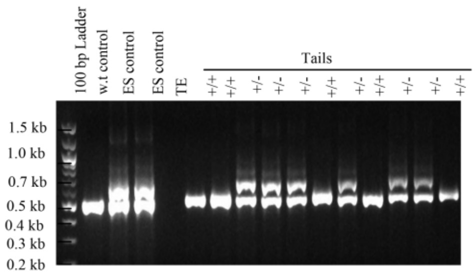

c

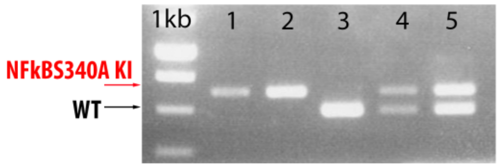

1. NFkB1 S340A knock in
2. NFkB1 S340A knock in
3. WT
4. Heterozygote
5. Heterozygote

Supplementary figure 6: Generation and genotyping of the *nfkb1*^{S340A} knock-in mouse.

(a) Diagram showing the strategy to generate the *nfkb1*^{S340A} knock-in mouse. (b) PCR conditions for genotyping the *nfkb1*^{S340A} mice and representative gel of the genotyping results during the generation of the line. (c) Representative gel of the genotyping results of in house *nfkb1*^{S340A} knock-in (red), WT (black) and heterozygous mice. Images a and b were generated at Taconic.

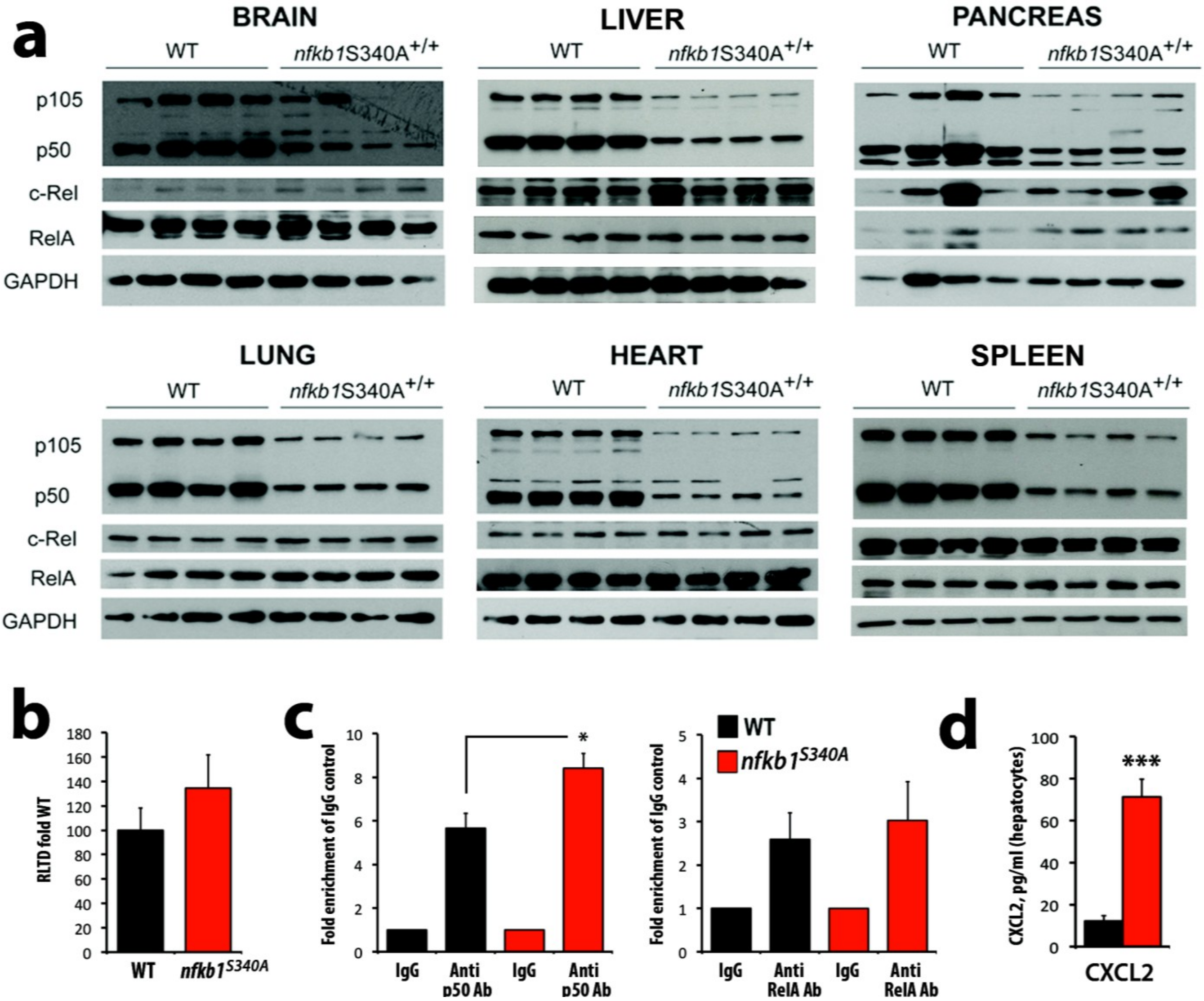

Supplementary figure 7: NF-κB subunit expression in tissues from the *nfkb1^{S340A}* mice.

(a) Western blots for p105/p50, c-Rel and RelA NF-κB subunits and GAPDH loading control in lysates isolated from WT and *nfkb1^{S340A}* brain, liver, pancreas, lung, heart and spleen. (b) p50 mRNA levels expressed as RLTD fold WT in liver from WT and *nfkb1^{S340A}* mice, n=3. (c) ChIP analysis of p50 and RelA recruitment to the S100A9 promoter in WT or *nfkb1^{S340A}* livers n=5. (d) CXCL2 ELISA performed on hepatocytes isolated from WT and *nfkb1^{S340A}* mice n=3. All data are means ± s.e.m. Statistical significance was determined using an unpaired t-test, $^{**}P < 0.01$ or $^{***}P < 0.001$ compared to WT control.

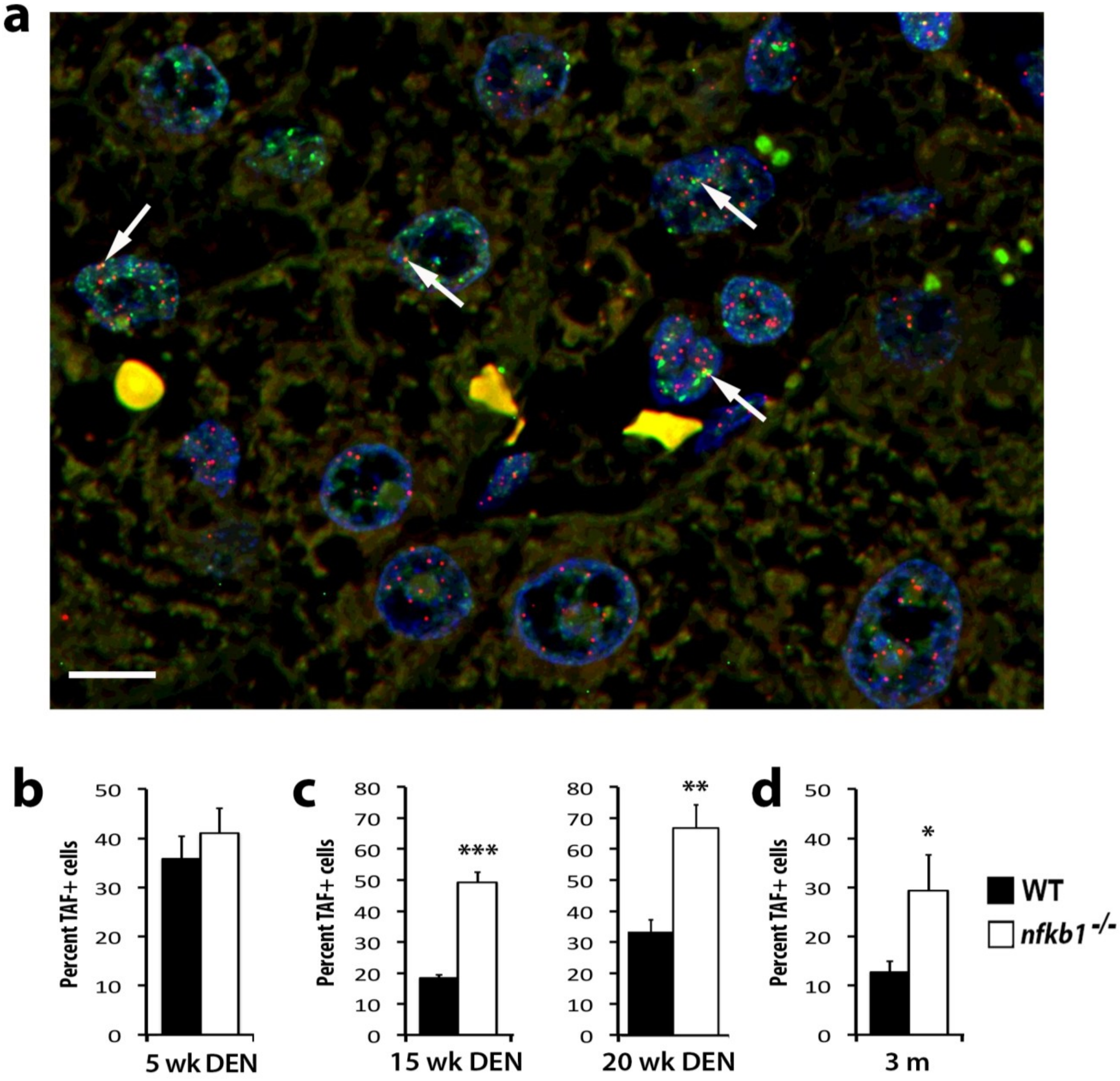

Supplementary figure 8: Accumulation of TAF+ hepatocytes is greater in *nfkb1-/-* mice. (a) Representative deconvolved maximum intensity projections of telomere FISH (red), phospho-H2A.X (γH2A.X – green), nuclei (DAPI - blue) staining in ALD. White arrows show a cluster of TAF+ hepatocytes, scale bar is 10 microns. (b-c) Graphs show percent TAF+ hepatocytes in 5 (b, n=3), 15 and 20 wk (c, n=5) DEN injured WT and *nfkb1-/-* mice. (d) Graph shows percent TAF+ hepatocytes in 3 month (n=3) uninjured WT and *nfkb1-/-* mice. All data are expressed as ± s.e.m. Statistical significance was determined using an unpaired t-test, $*P < 0.05$, $**P < 0.01$ or $***P < 0.001$ compared to WT control.

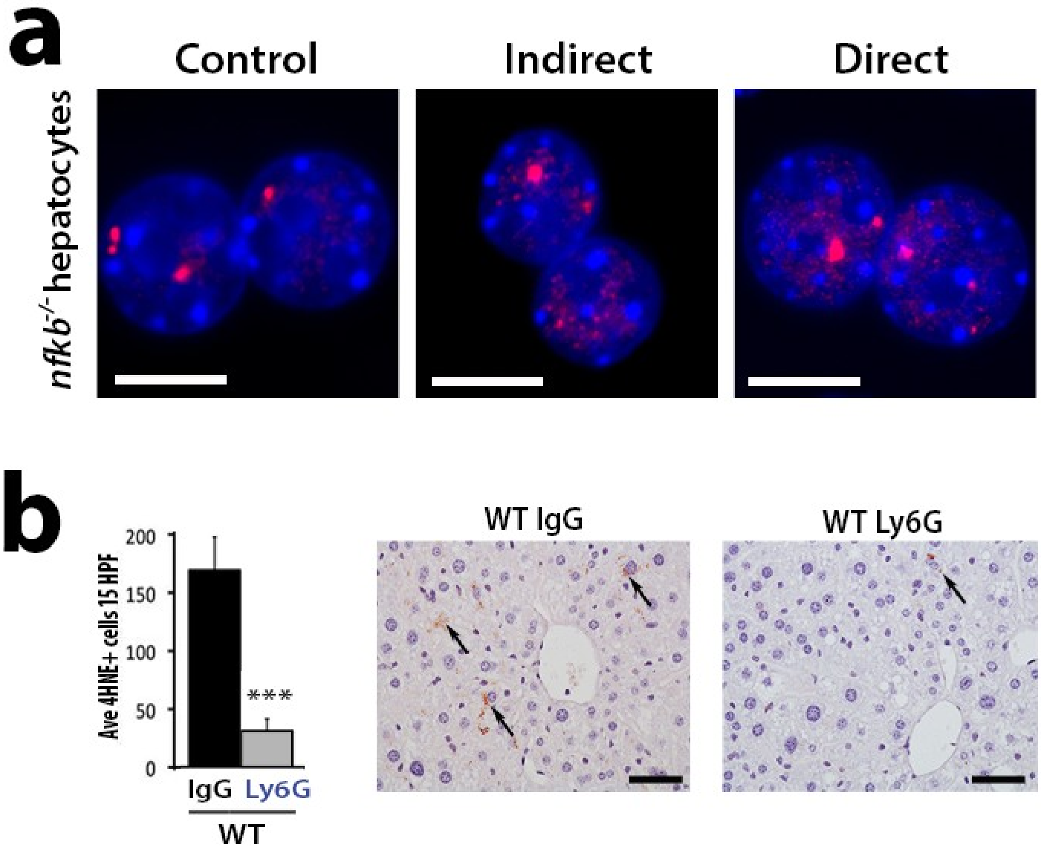

Supplementary figure 9: Neutrophils promote hepatocyte DNA damage and ROS induced lipid peroxidation in *nfkb1-/-* hepatocytes.

(a) Representative immunocytochemistry images and cell counts of DAPI/53BP1 stained *nfkb1-/-* hepatocytes only or *nfkb1-/-* hepatocytes in either direct or indirect (trans-well) co-culture with *nfkb1-/-* neutrophils n=3, scale bars are 10 microns. (b) Graph shows numbers of 4HNE+ hepatocytes in 40 wk DEN injured WT mice treated ± anti-Ly6G for 8 wks, n=6. Representative images at ×400 magnification of HNE stained livers from 40 wk DEN injured WT mice treated ± anti-Ly6G for 8 wks. Black arrows denote 4HNE+ stained hepatocytes, scale bars are 100 microns. Data are expressed as ± s.e.m. Statistical significance was determined using an unpaired t-test, ***$P < 0.001$ compared to IgG control.

Supplementary figure 10. Western blot.
Scans showing full size western blots for figure 3g, 3h, Supplementary figure 1d, Supplementary figure 2, Supplementary figure 5 and Supplementary figure 7 with molecular weight markers.

Figure 3g

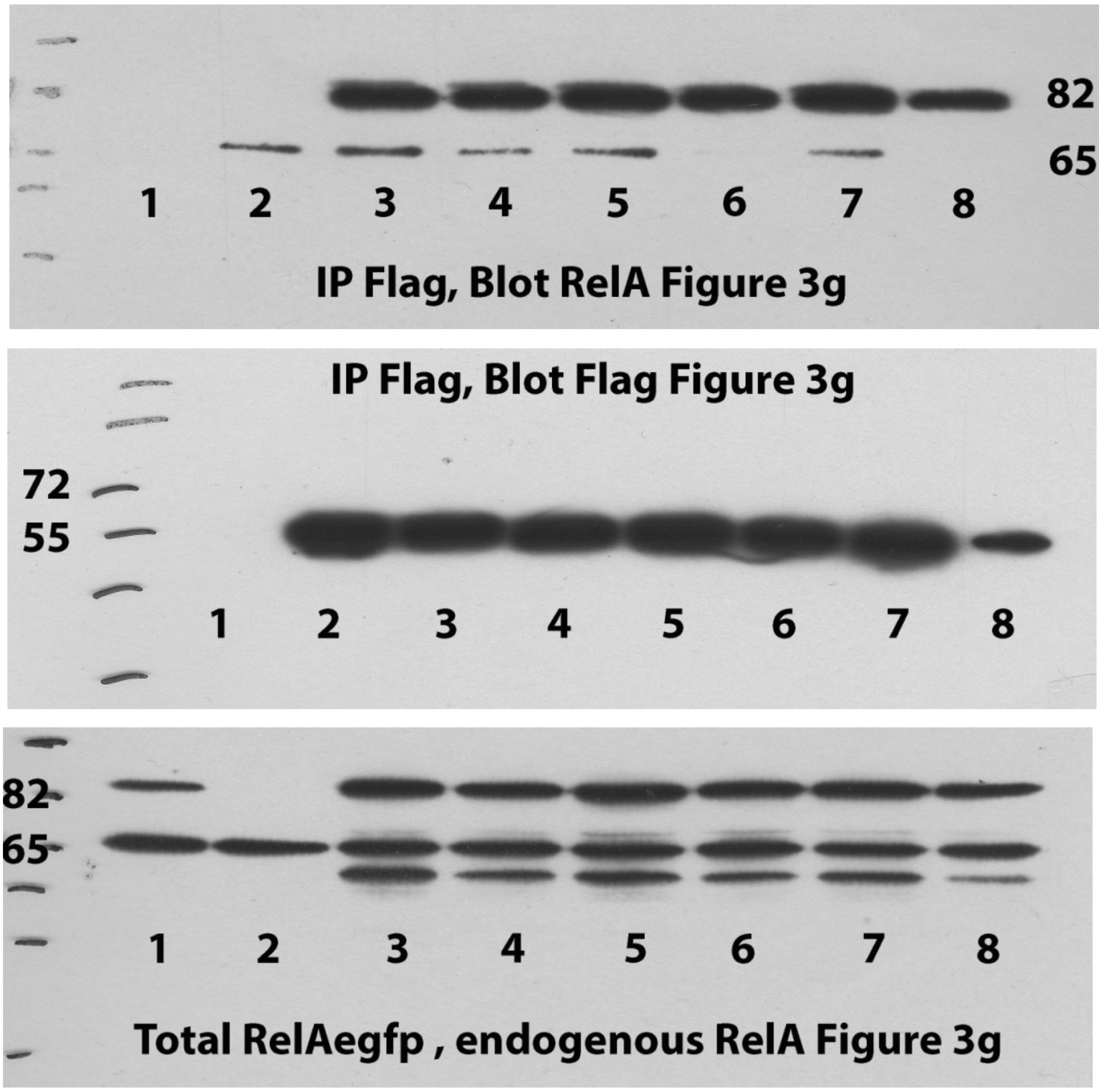

1. Control
2. Flag p50
3. Flag p50
4. Flag p50 T145A
5. Flag p50 S210A
6. Flag p50 S315A
7. Flag p50 S337A
8. Flag p50 S342A

Figure 3h

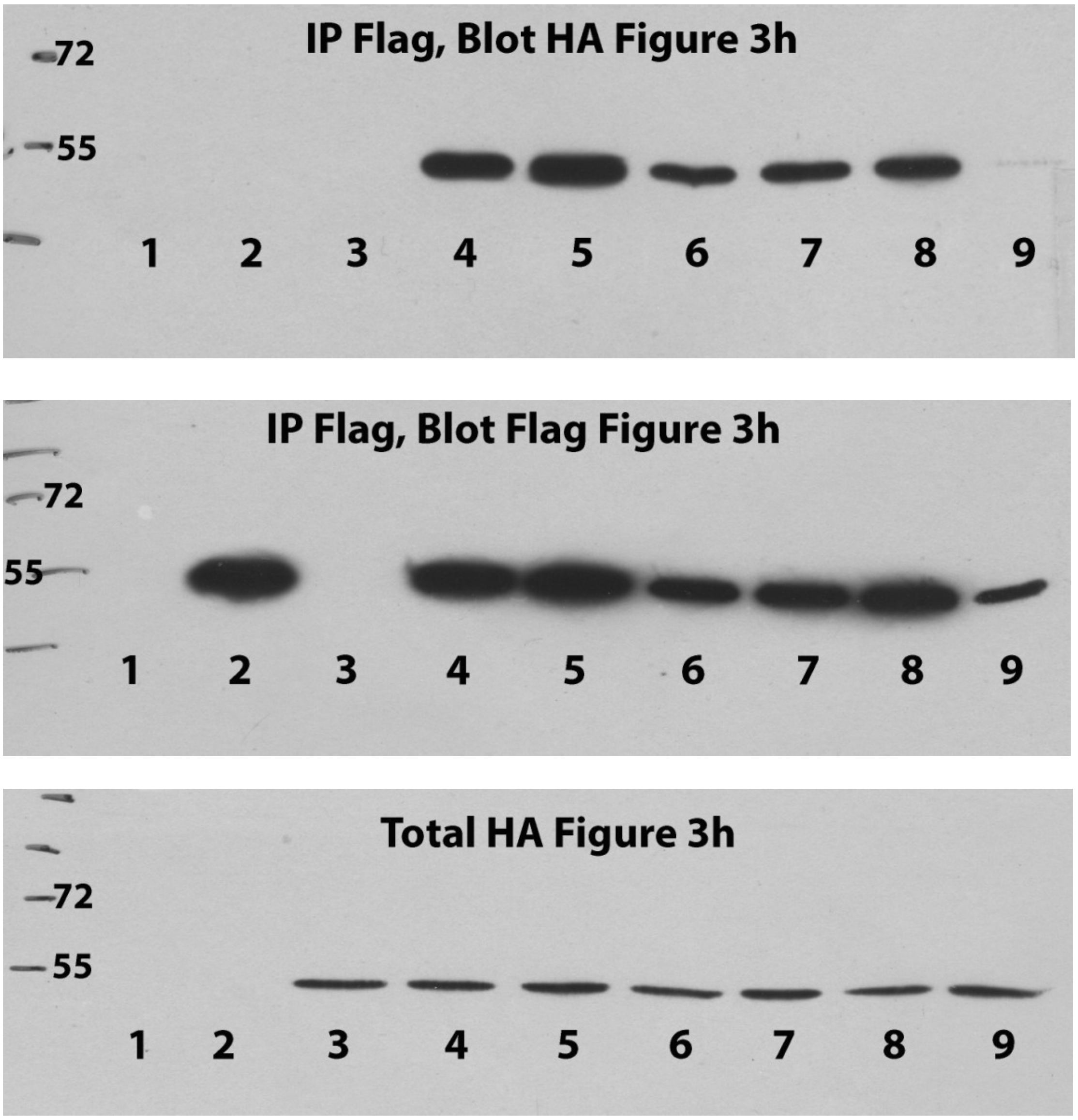

1. Control
2. Flag p50
3. HA p50
4. Flag p50
5. Flag p50 T145A
6. Flag p50 S210A
7. Flag p50 S315A
8. Flag p50 S337A
9. Flag p50 S342A

Supplementary figure 1d

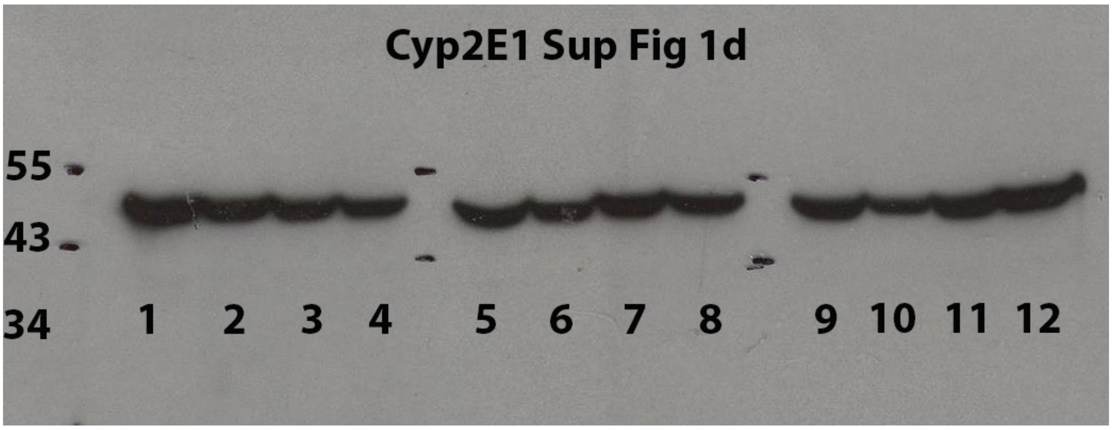

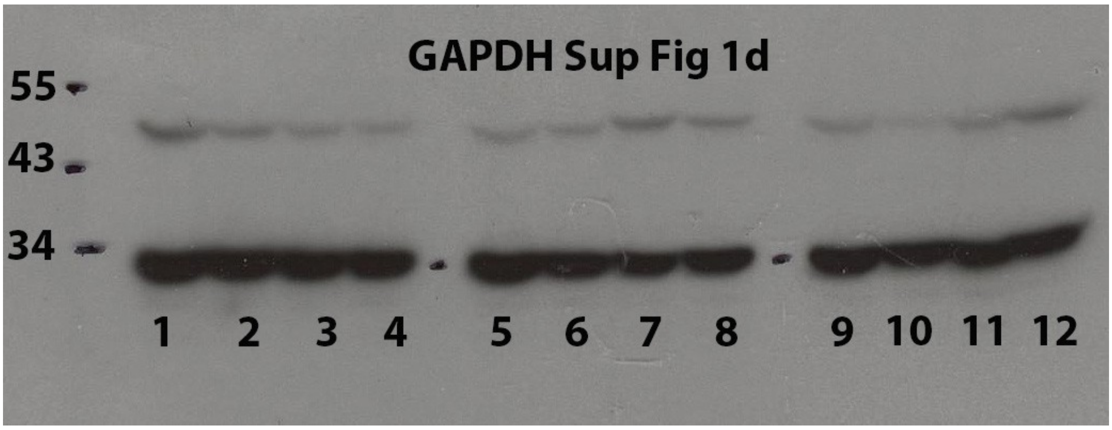

1 – 4. Wild type
5 – 8. Nfkb1-/-
9 – 12. Nfkb1 S340A+/+

Supplementary figure 2

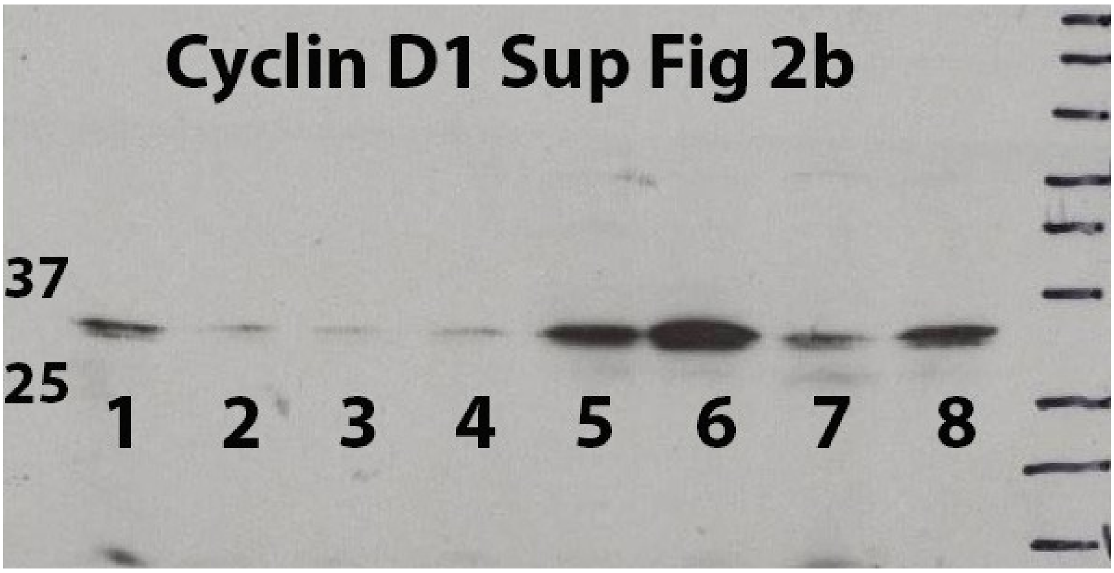

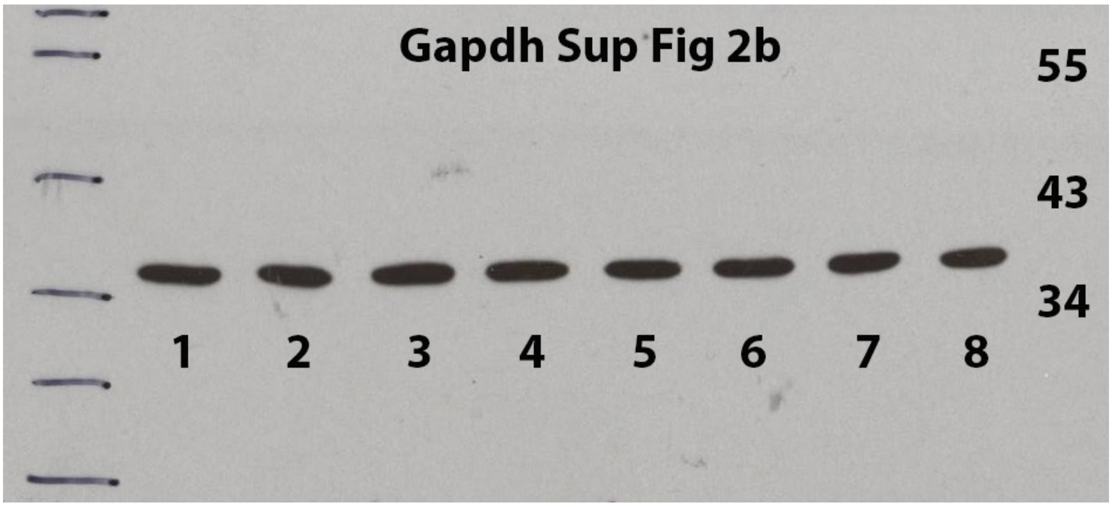

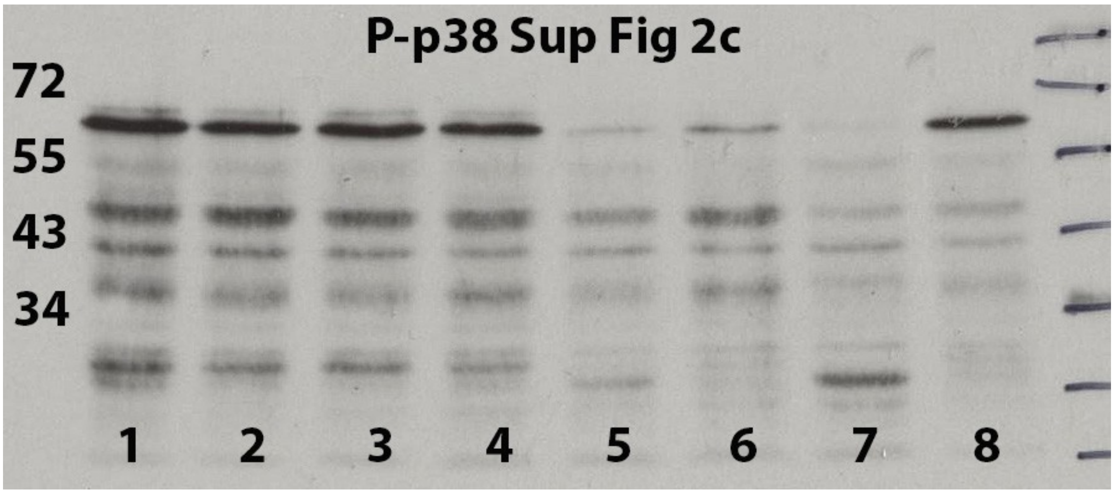

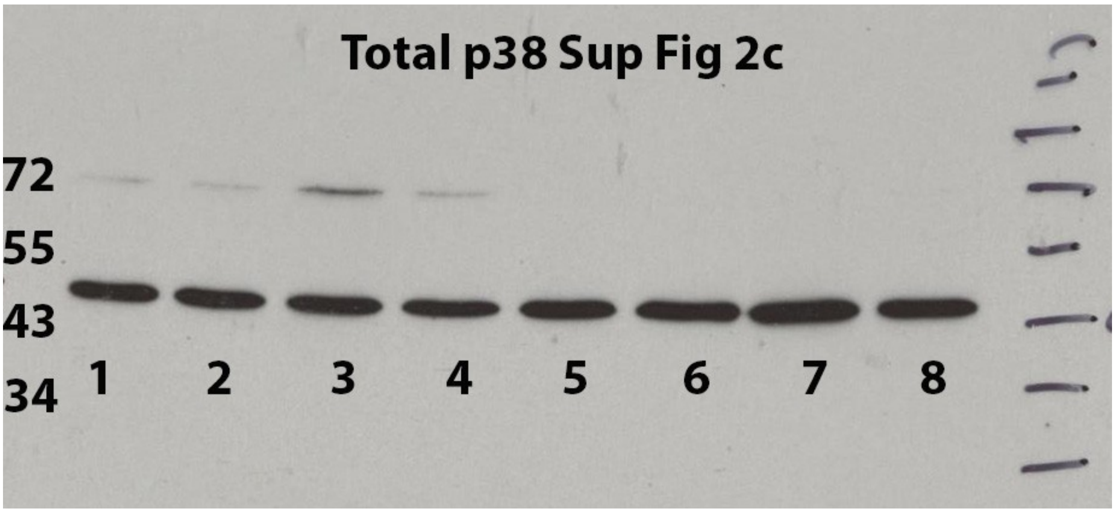

1 – 4. Wild type
5 – 8. Nfkb1-/-

Supplementary figure 5

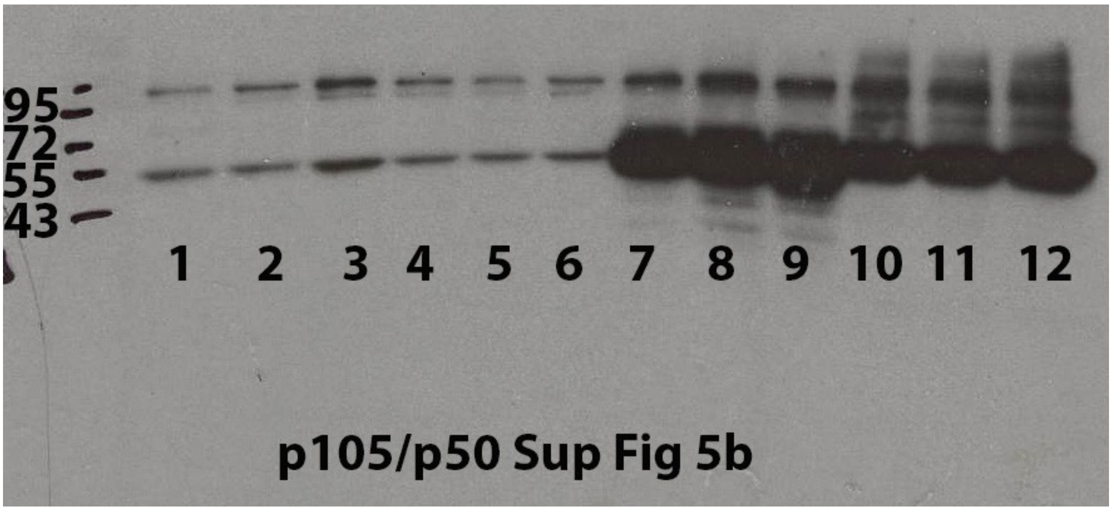

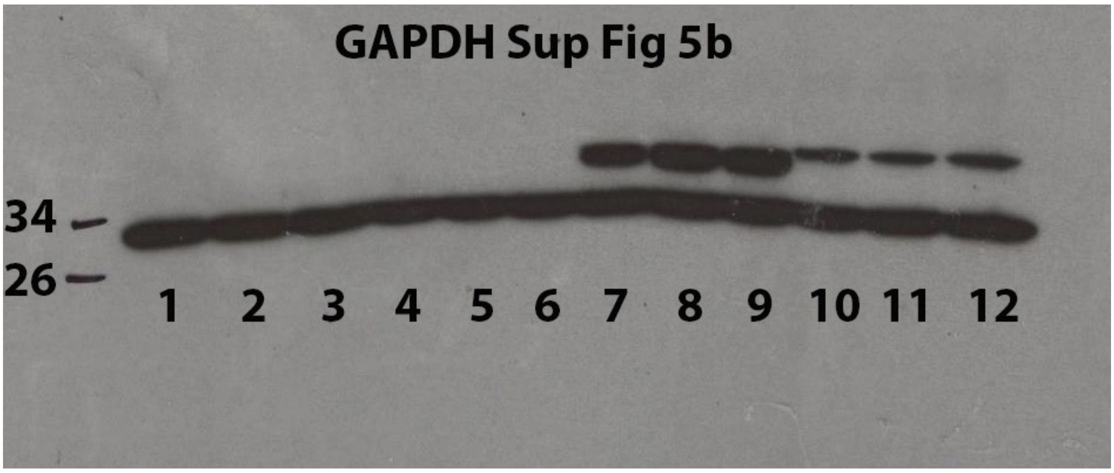

1 – 3. Media control
4 – 6. Control Adenovirus
7 – 9. p50 Adenovirus
10 - 12. p50 S340A Adenovirus.

Supplementary figure 7.

BRAIN

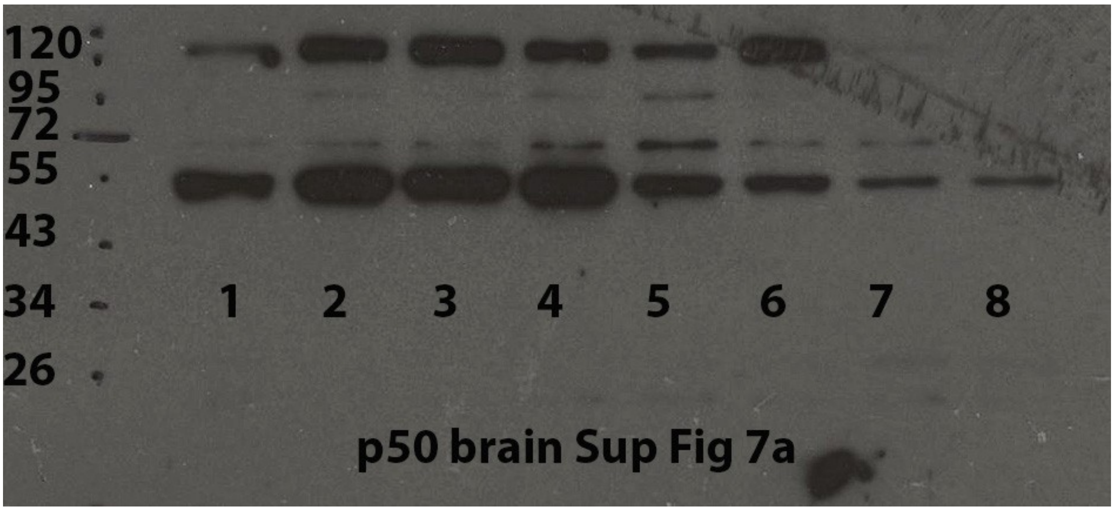

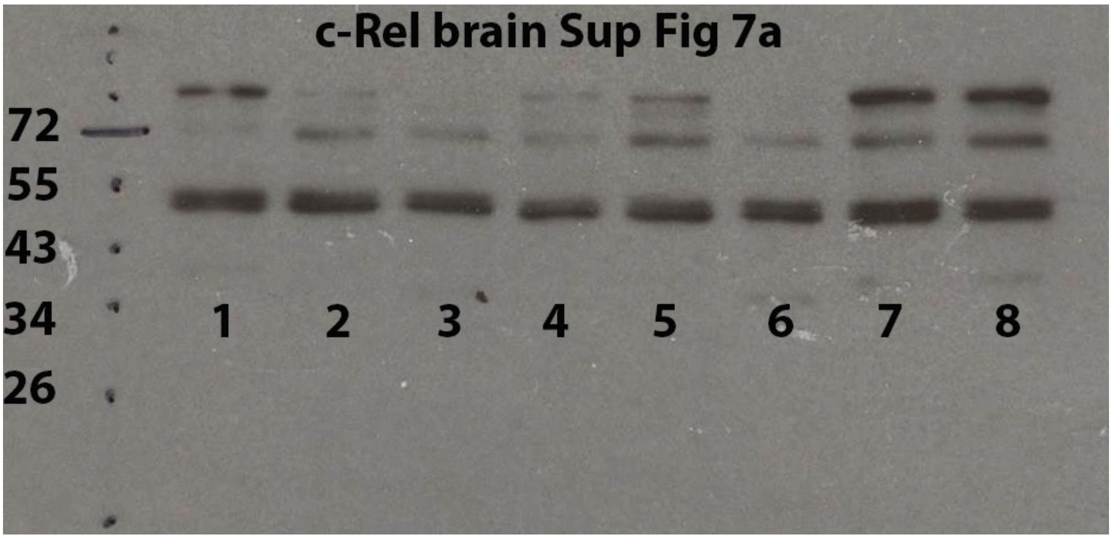

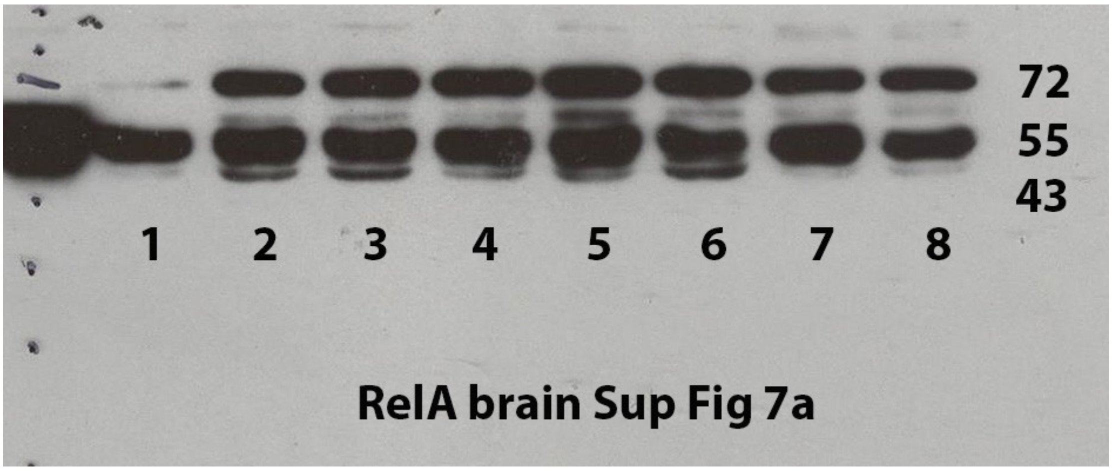

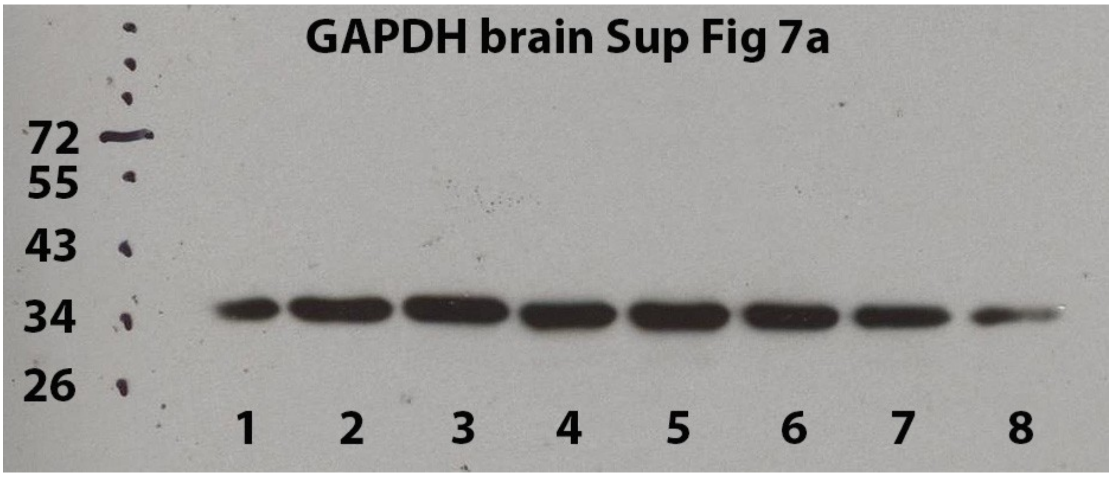
GAPDH brain Sup Fig 7a
72
55
43
34
26
1 2 3 4 5 6 7 8

LIVER

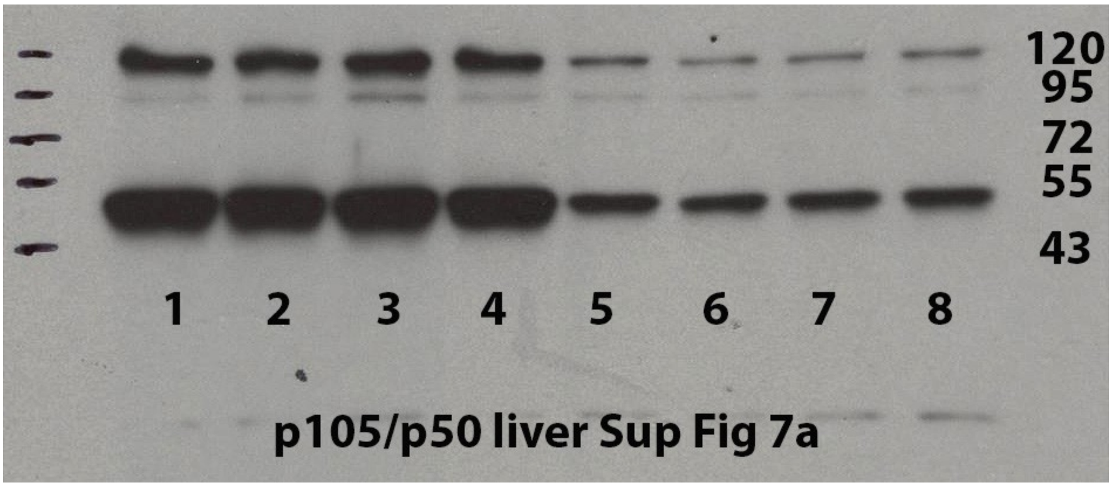
120
95
72
55
43
1 2 3 4 5 6 7 8
p105/p50 liver Sup Fig 7a

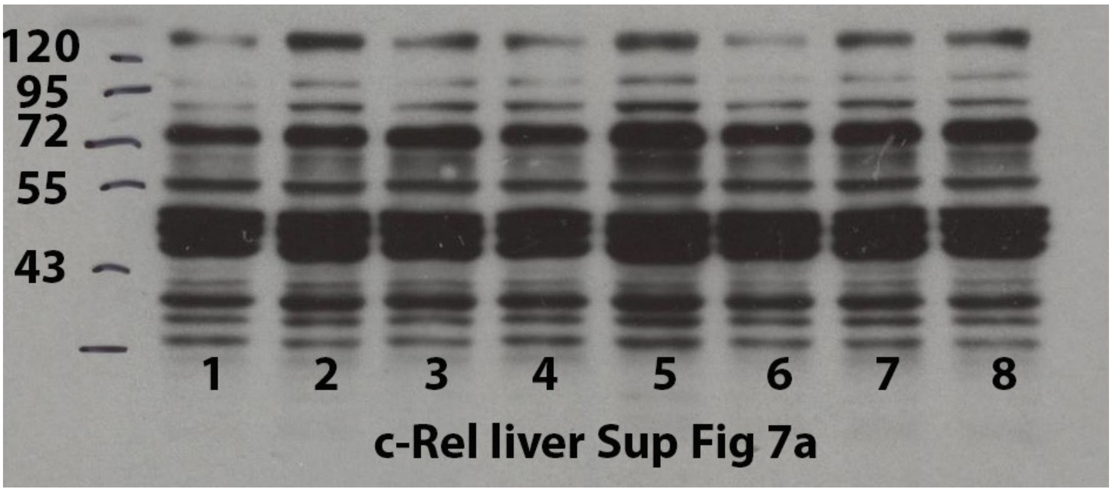
120
95
72
55
43
1 2 3 4 5 6 7 8
c-Rel liver Sup Fig 7a

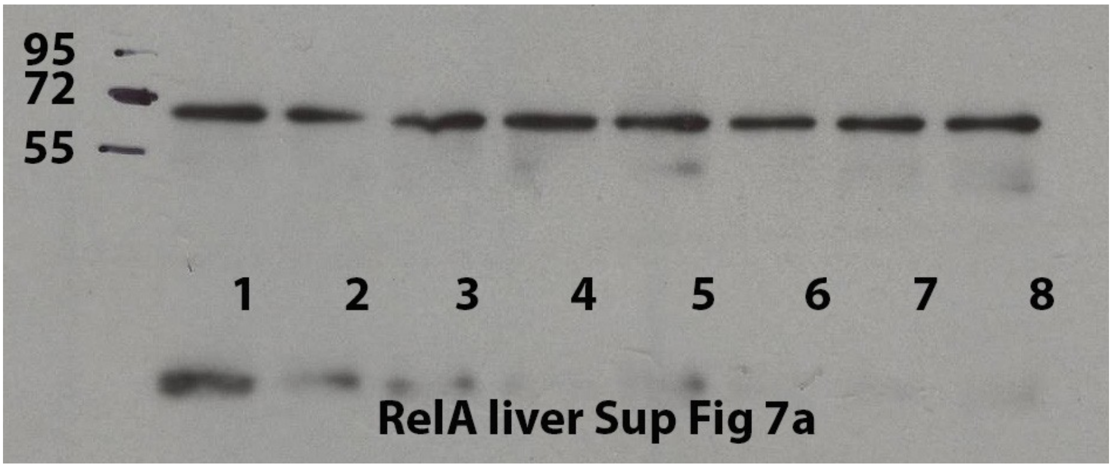
95
72
55
1 2 3 4 5 6 7 8
RelA liver Sup Fig 7a

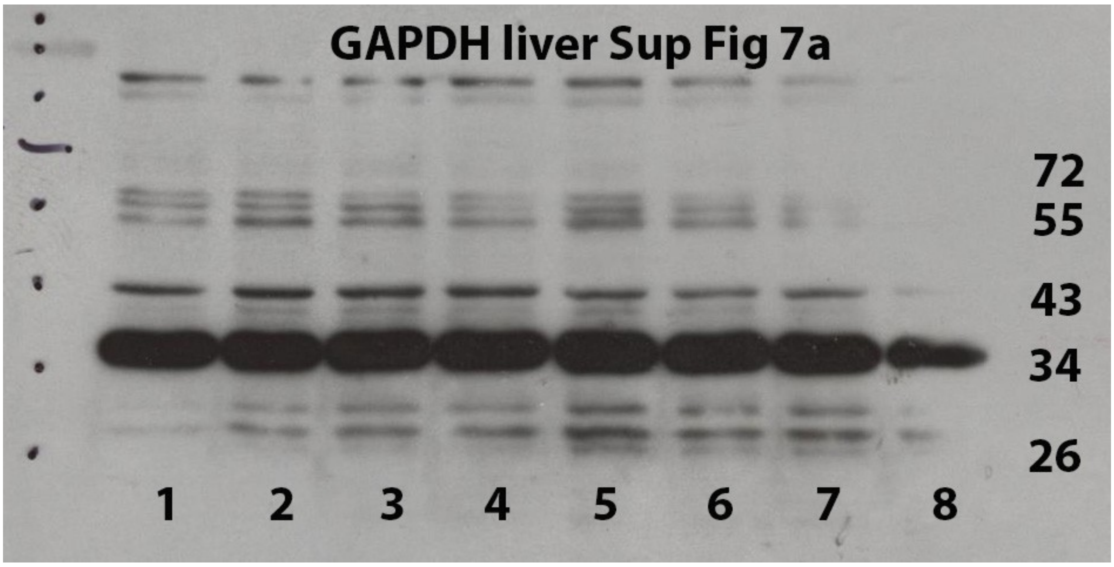
GAPDH liver Sup Fig 7a
72
55
43
34
26
1 2 3 4 5 6 7 8

PANCREAS

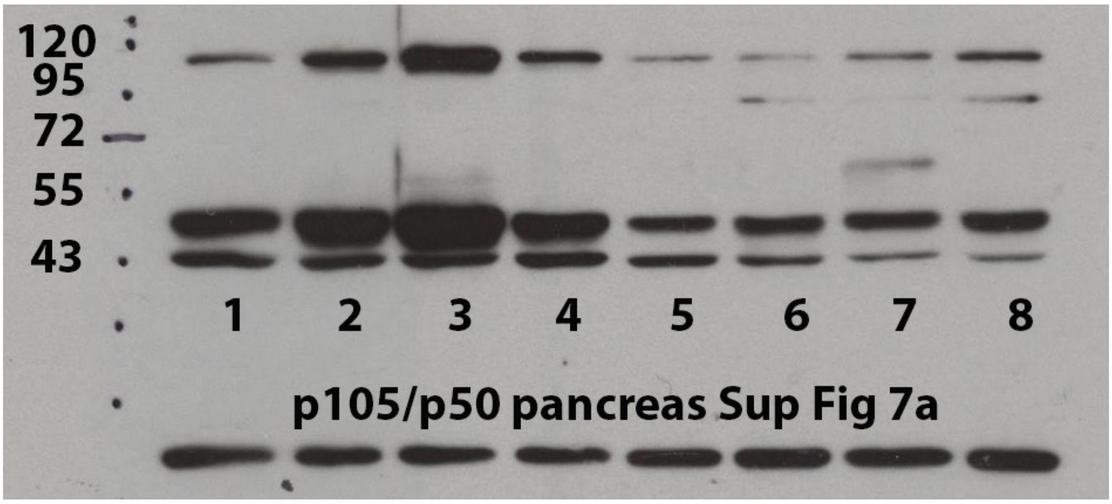
120
95
72
55
43
1 2 3 4 5 6 7 8
p105/p50 pancreas Sup Fig 7a

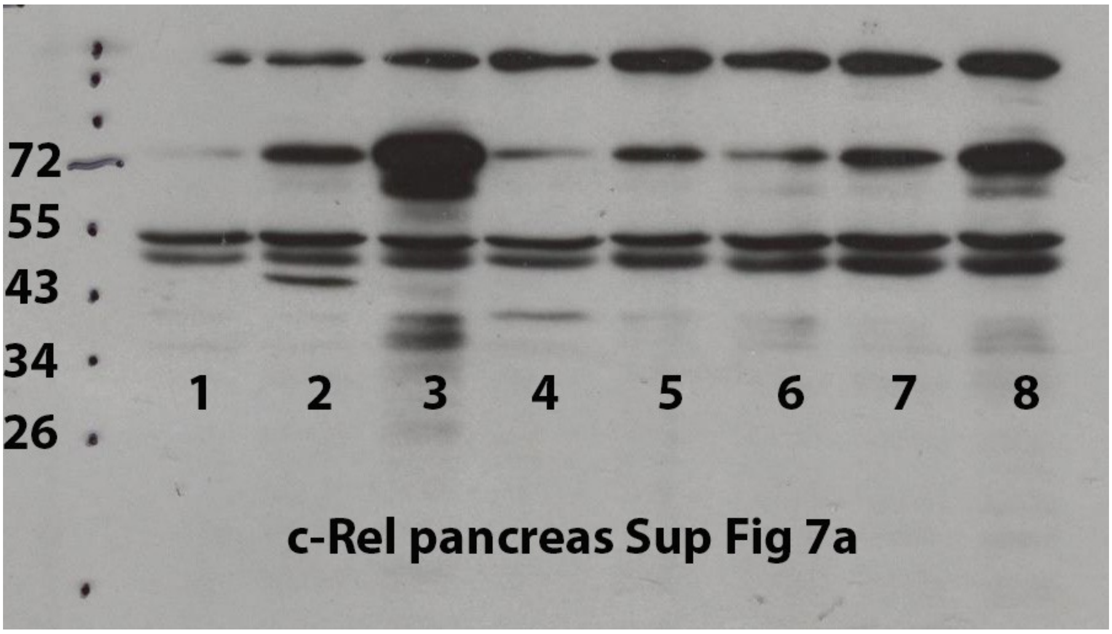
72
55
43
34
26
1 2 3 4 5 6 7 8
c-Rel pancreas Sup Fig 7a

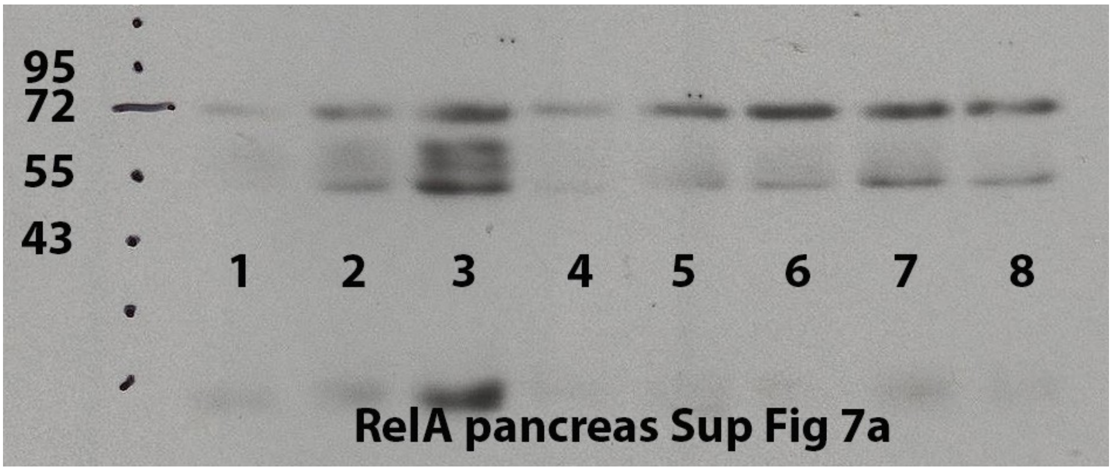
95
72
55
43
1 2 3 4 5 6 7 8
RelA pancreas Sup Fig 7a

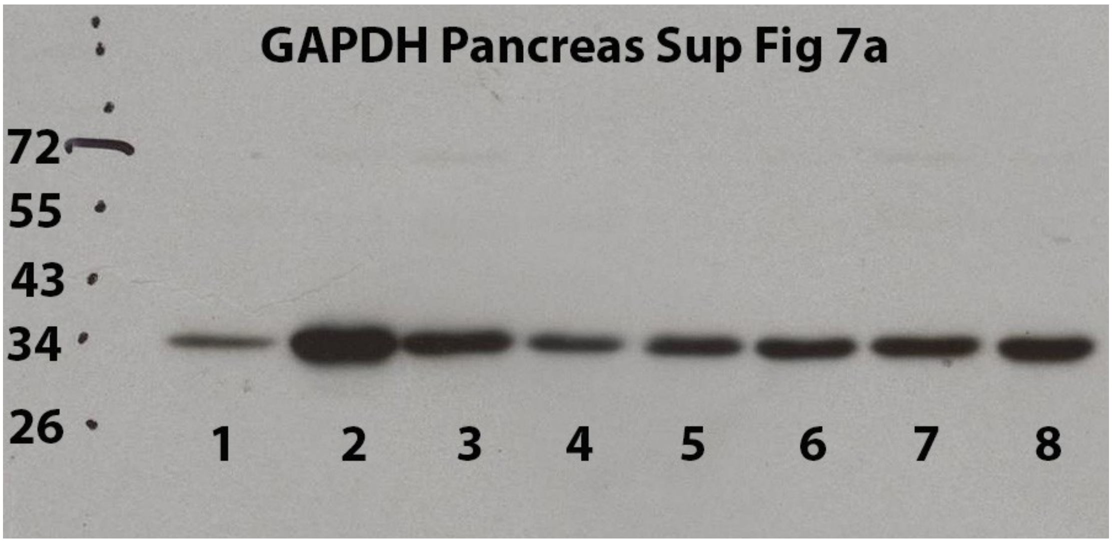
GAPDH Pancreas Sup Fig 7a
72
55
43
34
26
1 2 3 4 5 6 7 8

LUNG

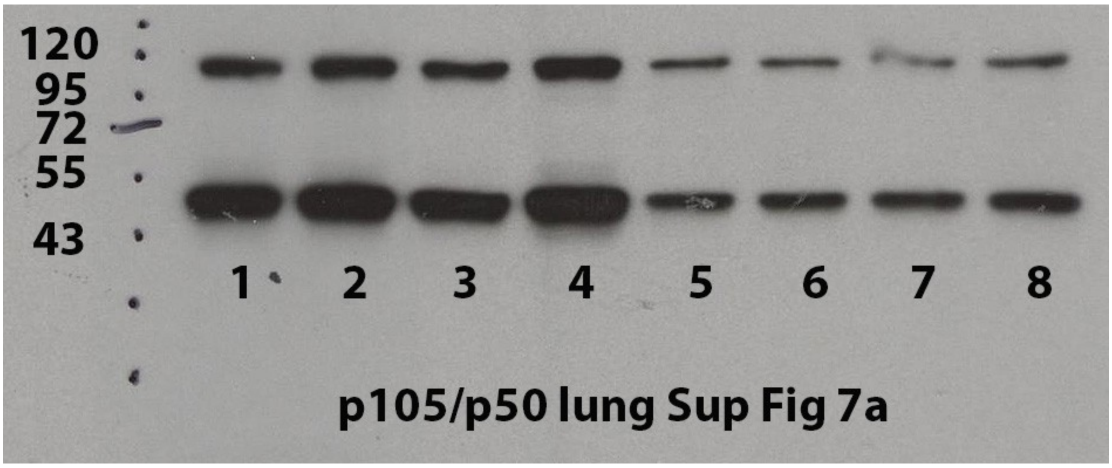
120
95
72
55
43
1 2 3 4 5 6 7 8
p105/p50 lung Sup Fig 7a

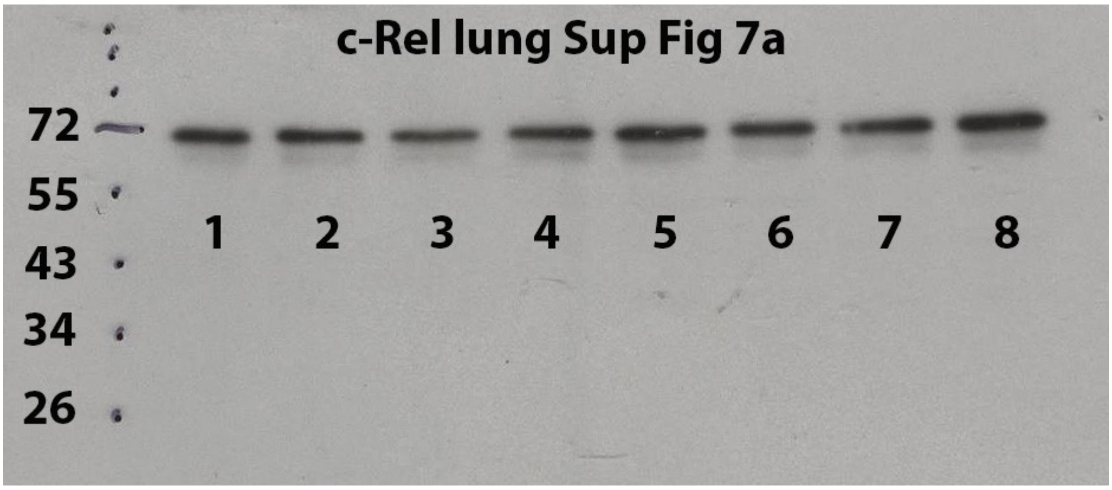
c-Rel lung Sup Fig 7a
72
55
43
34
26
1 2 3 4 5 6 7 8

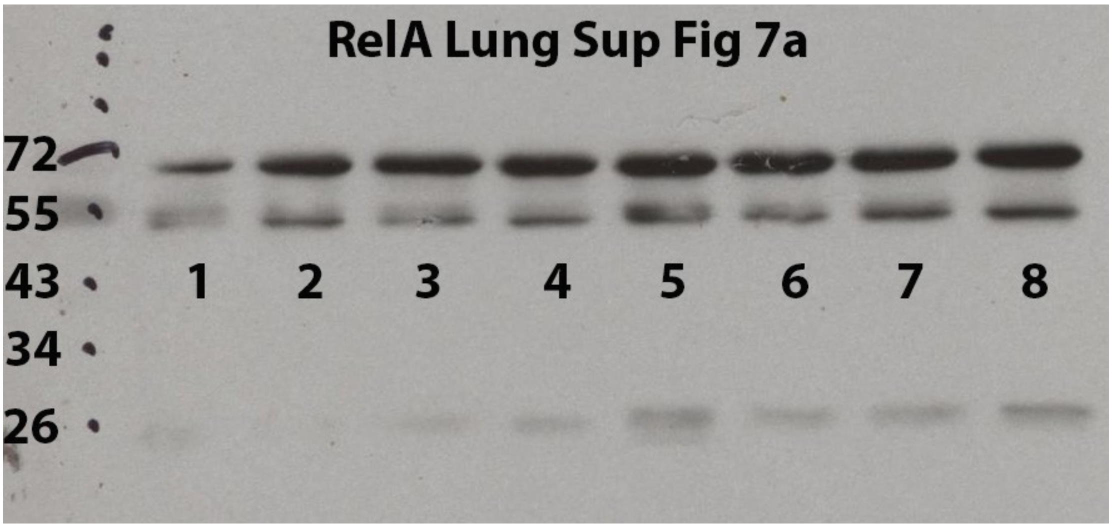
RelA Lung Sup Fig 7a
72
55
43
34
26
1 2 3 4 5 6 7 8

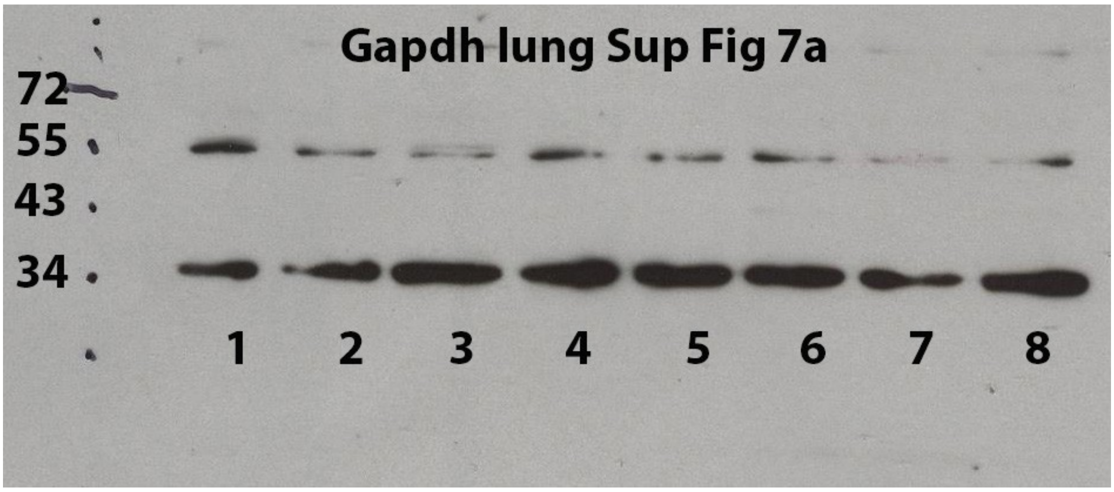
Gapdh lung Sup Fig 7a
72
55
43
34
1 2 3 4 5 6 7 8

HEART

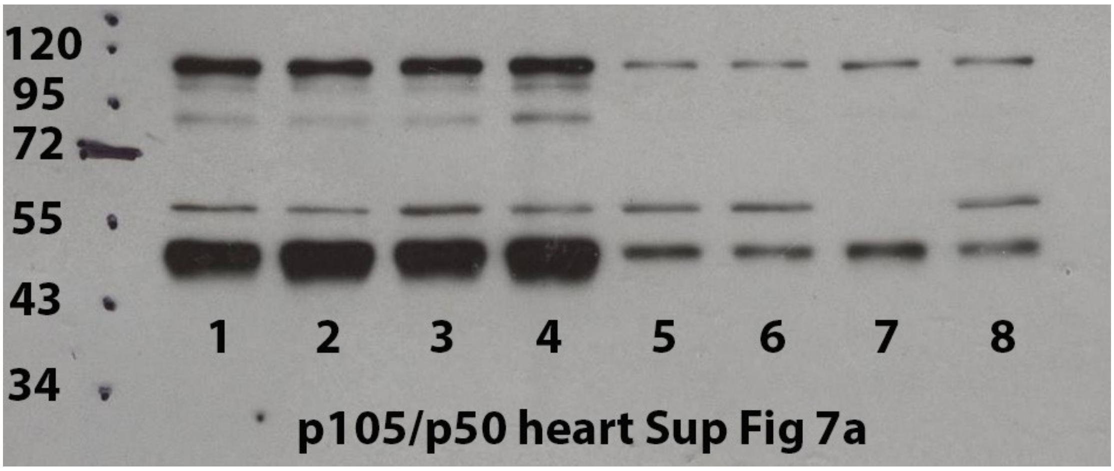
120
95
72
55
43
34
1 2 3 4 5 6 7 8
p105/p50 heart Sup Fig 7a

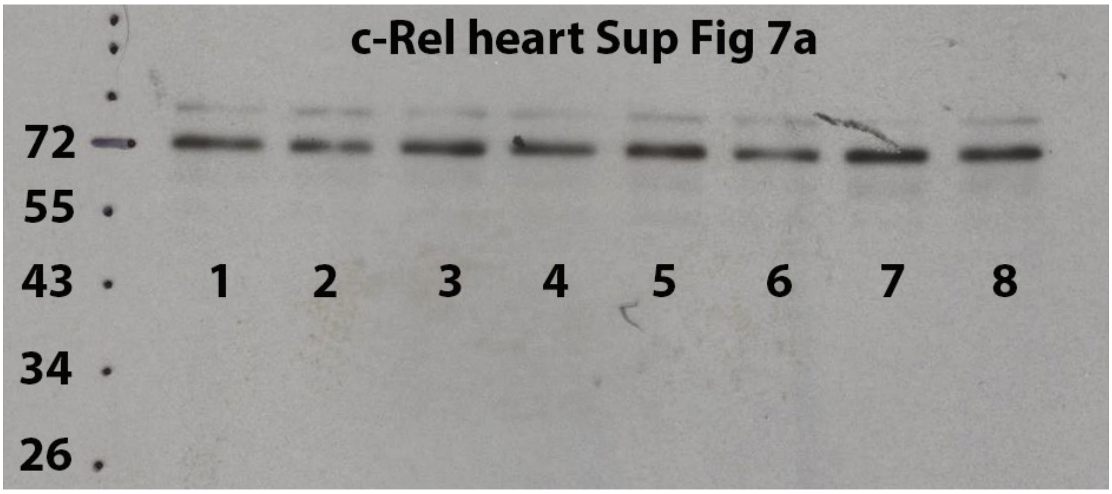
c-Rel heart Sup Fig 7a
72
55
43
34
26
1 2 3 4 5 6 7 8

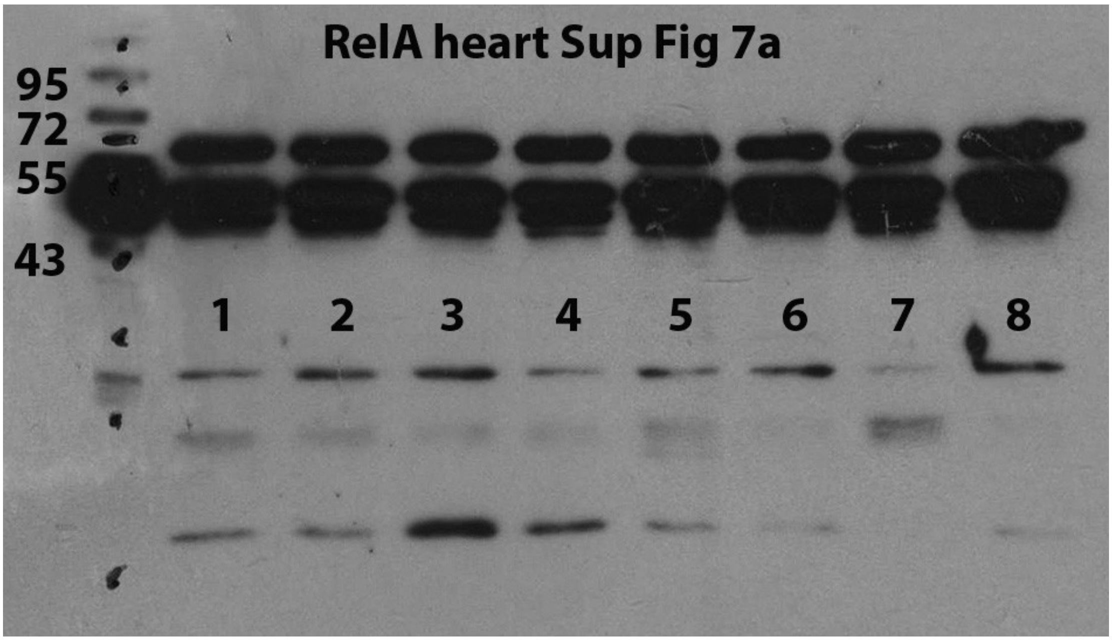
RelA heart Sup Fig 7a
95
72
55
43
1 2 3 4 5 6 7 8

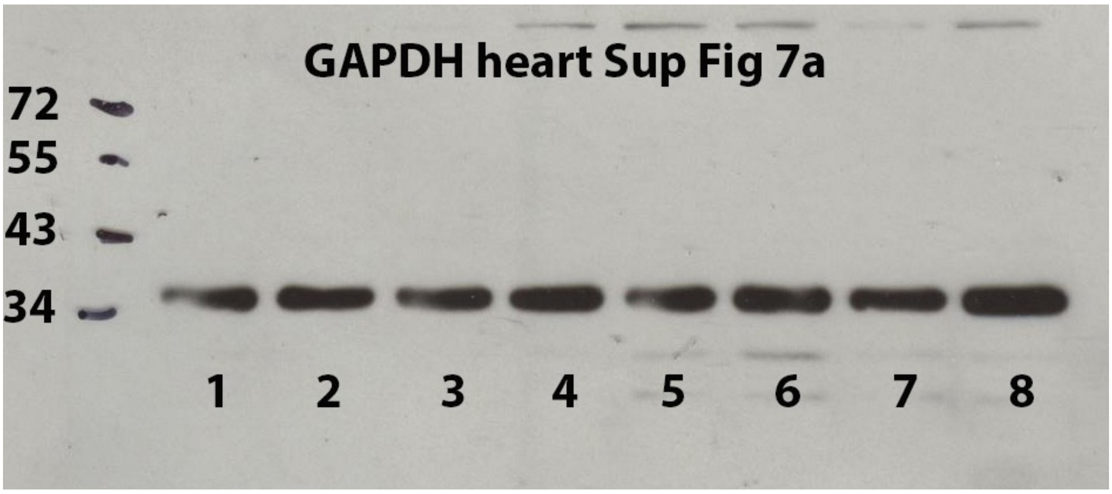
GAPDH heart Sup Fig 7a
72
55
43
34
1 2 3 4 5 6 7 8

SPLEEN

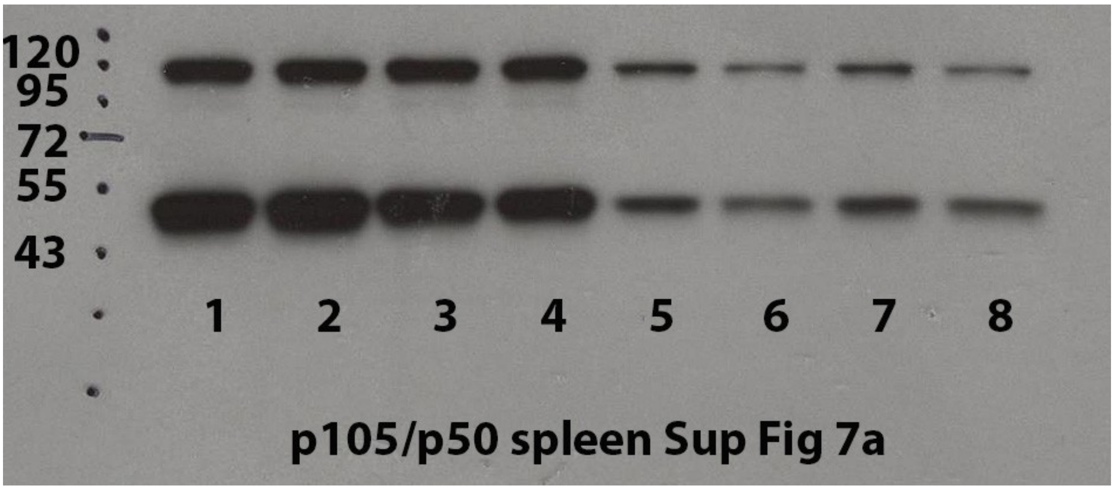
120
95
72
55
43
1 2 3 4 5 6 7 8
p105/p50 spleen Sup Fig 7a

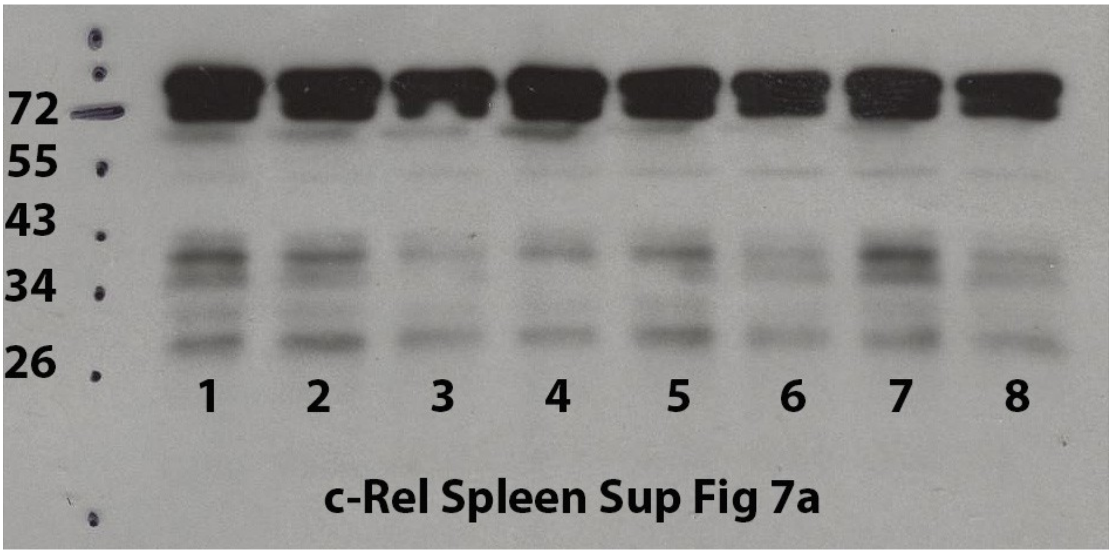

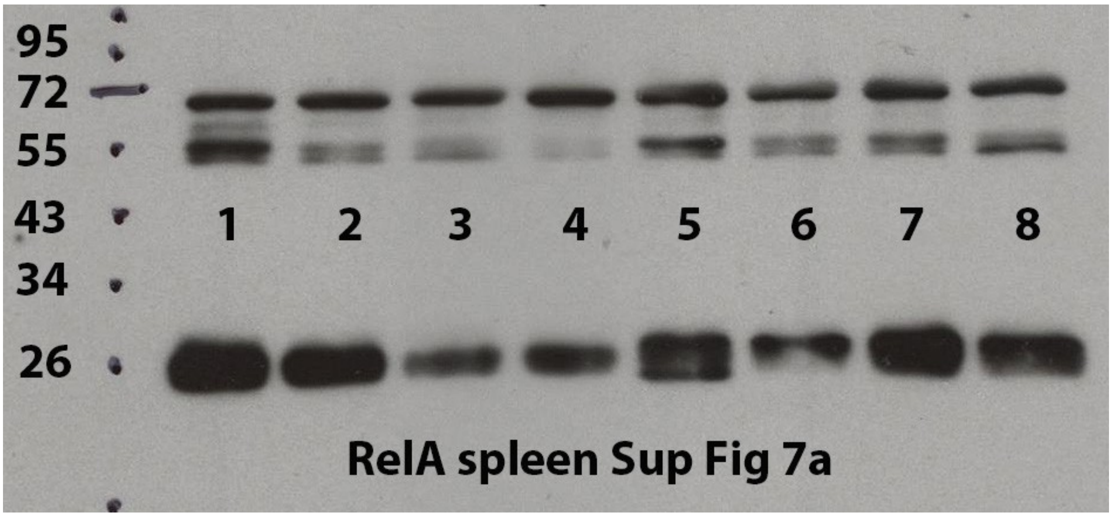

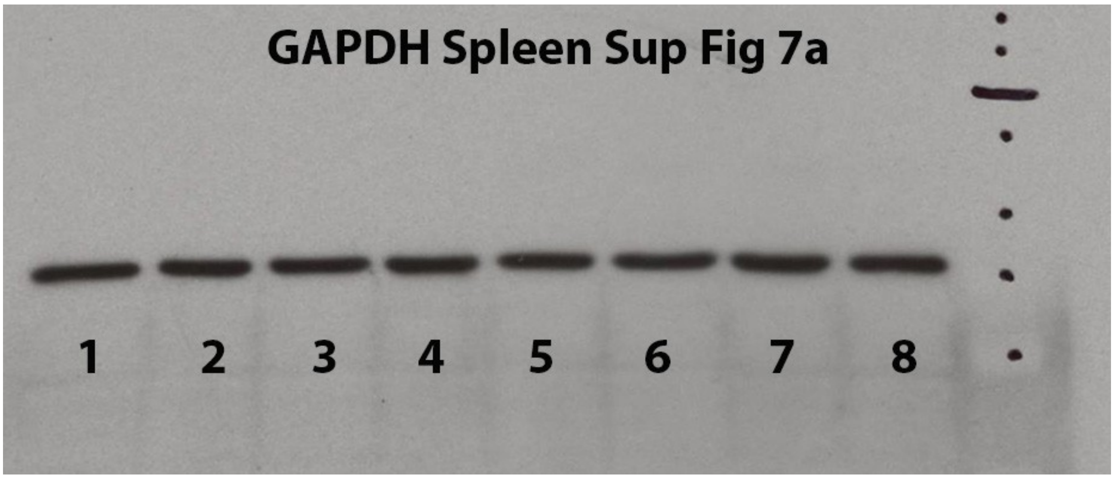

1 – 4. Wild Type
5 – 8. Nfkb1 S340A +/+

**Chronic DEN model**	**AVE ALT**	**P Value**	**AVE AST**	**P Value**
WT 20wk	55.0 ± 4.6	n/s	344.3 ± 27.8	n/s
Nfkb1-/- 20wk	88.7 ± 12.6		408.3 ± 71.3	
WT 30wk	128.1 ± 37.8	n/s	699.6 ± 144.6	n/s
Nfkb1-/- 30wk	117.3 ± 15.6		421.2 ± 51.6	
WT 40wk	218.1 ± 80.5	n/s	357.0 ± 73.4	n/s
Nfkb1-/- 40wk	227.4 ± 34.3		509.1 ± 93.2	
**Acute DEN model**	**AVE ALT**	**P Value**	**AVE AST**	**P Value**
WT	142.2 ± 13.2		723.0 ± 75.7	
Nfkb1-/-	272.4 ± 79.4	n/s	684.0 ± 119.6	n/s
Nfkb1 S340A$^{+/+}$	188.4 ± 29.4	n/s	649.8 ± 648	n/s

Table 1 Liver transaminases

Serum alanine transaminase (ALT) and aspartate aminotransferase (AST) levels in chronic DEN in WT and nfkb1$^{-/-}$ livers and acute DEN in WT, nfkb1$^{-/-}$ and S340A$^{+/+}$ livers, data is average ± s.e.m for n= 9 mice and n=5 mice per genotype for the chronic and acute studies respectively. P values were calculated using an unpaired T-Test.

Marker	WT	Nfkb1$^{-/-}$	P Value
Sirius red	0.702 ± 0.05	1.27 ± 0.23	p= 0.0349 *
αSMA	2.73 ± 0.19	12.6 ± 1.27	p=0.0006 ***
K19	0.42 ± 0.041	3.64 ± 0.29	p= 0.0145 **
F4/80	4.31 ± 0.80	4.26 ± 0.87	n/s
NE	0.91 ± 0.26	12.55 ± 3.23	p=0.0156 **
NIMP1	1.34 ± 0.33	9.90 ± 1.64	p=0.0036 **
S100A9	2.04 ± 0.19	13.15 ± 1.76	p=0.0008 ***
CD3	4.80 ± 0.25	63.60 ± 7.07	p=0.0004 ***
PCNA	0.98 ± 0.25	7.65 ± 1.87	p=0.0251 *
γH2Ax	0.63 ± 0.13	9.88 ± 1.37	p=0.0023 **
Collagen IV	16.42 ± 1.03	11.78 ± 0.77	P= 0.01 **

Table 2 Histology analysis of ageing mice

Measurement of markers of fibrosis, inflammation, ductular reaction, proliferation and DNA damage in 20 month old WT and nfkb1$^{-/-}$ livers data is average ± s.e.m for n= 5 WT and 10 nfkb1$^{-/-}$ mice per genotype. P values were calculated using an unpaired T-Test.

nfkb1 mice	HCC	Dysplasia Or Adenoma	Diffuse GS	Broad trabeculae	Mitoses	N/C ratio	Focal reticulin loss	Fibrosis (stage)	Steatosis (grade)	Inflam (grade)	Ballooning	Granuloma	Ductular proliferation
**1**	YES	NO	NO	NO	NO	NO	NO	YES (3)	YES (1)	YES (2)	NO	NO	YES
**2**	NO	NO	NO	NO	NO	NO	NO	YES (3)	YES (1)	YES (2)	NO	NO	YES
**3**	YES	YES	NO	YES	NO	YES	YES	YES (2)	NO	YES (2)	NO	NO	NO
**4**	NO	YES	NO	NO	NO	NO	NO	YES (2)	YES (3)	YES (2)	YES	YES (lipo-granuloma)	YES
**5**	YES	NO	YES	YES	YES	YES	YES	NO	NO	YES	NO	NO	YES
**6**	NO	YES<br>2 HCA	NO	NO	NO	NO	NO	YES (3)	YES (2)	YES (1)	NO	YES (portal)	NO
**7**	NO	NO	NO	NO	NO	NO	NO	YES (3)	NO	YES (3)	NO	NO	YES
**8**	NO	NO	NO	NO	NO	NO	YES	YES (3)	NO	YES (2)	NO	NO	YES
**9**	NO	NO	NO	NO	NO	NO	YES	YES (1)	NO	YES (1)		NO	YES (focal)
**10**	NO	NO	NO	NO	NO	NO	NO	YES (2)	YES	YES (2)	YES	NO	YES
**WT Mice**													
**1**	NO	NO	NO	NO	NO	NO	YES	NO	Mild	YES PI<br>NO LI	YES (focal)	NO	NO
**2**	NO	NO	NO	NO	NO	NO	YES	NO	Mild	YES PI<br>NO LI	YES	NO	NO
**3**	NO	NO	NO	NO	NO	NO	YES	NO	Mild	YES PI<br>NO LI	YES (focal)	NO	NO
**4**	NO	NO	NO	NO	NO	NO	YES	NO	Mild	YES PI<br>NO LI	YES (focal)	NO	NO
**5**	NO	NO	NO	NO	NO	NO	YES	NO	Mild	YES PI	YES (focal)	NO	NO

Table 3 Histological characterisation of the features of chronic liver disease (CLD)

Spontaneous development of adenoma and hepatocellular carcinoma (HCC) in 20 month old WT and nfkb1$^{-/-}$ livers. *NB – nfkb1 -/- mice 2 and 4 developed a (1 HCA & 1biliary microhamartoma/bile duct adenoma)*

Group	CXCL1	CXCL2	S100A9	TNFα
Uninjured mice - ageing				
WT 3m	1.0 ± 0.39	1.0 ± 0.57	1.0 ± 0.33	-
Nfkb1$^{-/-}$ 3m	0.75 ± 0.51	2.29 ± 1.05	6.67 ± 0.45	-
WT 9m	0.03 ± 0.002	1.99 ± 0.16	0.74 ± 0.13	-
Nfkb1$^{-/-}$ 9m	0.33 ± 0.12 ***	3.04 ± 0. 38 *	6.19 ± 4.34 *	-
WT 12m	0.069 ± 0.01	1.13 ± 0.54	0.45 ± 0.1	-
Nfkb1$^{-/-}$ 12m	0.19 ± 0.05 ***	9.0 ± 4.5 **	1.43 ± 0.5 *	-
WT 15m	0.07 ± 0.02	1.14 ± 0.3	0.93 ± 0.38	-
Nfkb1$^{-/-}$ 15m	1.45 ± 0.03 ***	3.61 ± 1.0 *	4.73 ± 0.98 *	-
20 week DEN model				
WT	1.0 ± 0.15	1.0 ± 0.23	1.0 ± 0.16	1.0 ± 0.12
Nfkb1$^{-/-}$	4.96 ± 0.1 ***	9.45. ± 3.3.6 *	31.55 ± 10.99. **	4.64 ± 0.82 ***
30 week DEN model				
WT NT	1.0 ± 0.32	1.0 ± 0.25	1.0 ± 0.72	1.0 ± 0.18
WT T	2.10 ± 0.91	1.0 ± 0.23	0.34 ± 0.15	1.34 ± 0.41
Nfkb1$^{-/-}$ NT	9.27 ± 3.07**	7.14 ± 1.59 *	10.71 ± 3.48*	11.51 ± 3.50**
Nfkb1$^{-/-}$ T	6.95 ± 1.72*	6.49 ± 1.70 **	16.40 ± 5.73*	11.51 ± 4.30**
40 week DEN model				
WT NT	1.0 ± 0.23	1.0 ± 0.52	1.0 ± 0.46	1.0 ± 0.51
WT T	4.64 ± 1.9 *	0.26 ± 0.07	0.95 ± 0.38	1.13 ± 0.19 *
Nfkb1$^{-/-}$ NT	2.35 ± 0.27	3.36 ± 0.87	3.0 ± 0.78	4.57 ± 0.80
Nfkb1$^{-/-}$ T	7.65 ± 1.81 **	0.83 ± 0.28	7.61 ± 1.18 **	7.58 ± 1.40 ***
S100A9$^{-/-}$ NT	0.45 ± 0.10	0.49 ± 0.19	0	0.35 ± 0.04
S100A9$^{-/-}$ T	4.33 ± 1.55 **	0.29 ± 0.01	0	1.63 ± 0.34

Table 4 Hepatic cytokines are elevated in normal and injured WT and nfkb1-/- mice Hepatic cytokine mRNA expression in normal (uninjured mice), 20 (non-tumor) and 30 and 40 wk (Tumor V's Non-Tumor) DEN in WT and nfkb1-/- livers. Data are expressed as average fold increase in RLTD compared to WT ± s.e.m. for n=4-5 (uninjured mice), n=8 (20 week DEN), n=5 (30 week DEN) and n=6 (40 week DEN) mice per genotype. P values were calculated using an unpaired T-Test.

Gene	Forward Primer (5'-3')	Reverse Primer (5'-3')	Annealing temperature °C
mGAPDH	GCACAGTCAAGGCCGAGAAT	GCCTTCTCCATGGTGGTGAA	55
mS100A9	CACCCTGAGCAAGAAGGAAT	TGTCATTTATGAGGGCTTCATTT	55
mS100A8	TGCGATGGTGATAAAAGTGG	GGCCAGAAGCTCTGCTACTC	60
mTNF-α	GACCAGGCTGTCGCTACATCA	CGTAGGCGATTACAGTCACGG	60
mIL-6	GAGGATACCACTCCCAACAGA	AAGTGCATCATCGTTGTTCATA	60
mIL-1β	GCAACTGTTCCTGAACTCAACT	ATCTTTTGGGGTCCGTCAACT	60
mCXCL1	CTGGGATTCACCTCAAGAACATC	CAGGGTCAAGGCAAGCCTC	60
mCXCL2	CCAACCACCAGGCTACAGG	GCGTCACACTCAAGCTCTG	60
mp50	TGGCTTTGCAAACCTGGGAA	AATACACGCCTCTGTCATCCGT	60
**ChIP primers**	**Forward Primer (5'-3')**	**Reverse Primer (5'-3')**	**Annealing temperature °C**
mS100A9	TATGCTGGTGAGGATGTGGA	TGGGTTCTTTCCAGCTTCTG	58
mCXCL1	GTTGGCAAAAGCAAACCACC	ACTACAGTGATTTGCGGGGA	55
mCXCL2	GACATCCCAGGGTCCCATAG	TGCACGATGTCTGGAAAAGC	60

Table 5
Table of primer sequences and annealing temperatures used for mouse quantitative RT-PCR and ChIP promoter quantitative PCR reactions.

Flag p50 mutant primers	Forward primer 5' – 3'	Reverse primer 5' – 3'
1st reaction common Flag-p50 forward (Hind III)	*aagctt*accatggactacaaggacgatgacaag**atg**gcagaagatgatcca	
**HA p50 mutant primers**		
1st reaction common HA-p50 forward (Hind III)	ctcc*aagctt*aaaatgtacccatacgacgtcccagactacgct**atg**gcagaagatgatccatat	
p50T145AR	cttcatgtg**gct**aagaaaaaagtatttgaaacactg	cagtgtttcaaatacttttttctt**agc**cacatgaag
p50S210AR	aggagatggacctc**gct**gtggt**a**cggctcatgttta	taaacatgagccg**t**accac**agc**gaggtccatctcct
p50T315AR	ttgccattgtcttcaaa**gct**ccaaagtataaagata	taatatctttatactttgg**agc**tttgaagacaatgg
p50S337AR	tttgt**t**ca**a**cttcggaggaaa**gct**gacttggaaact	gtttccaagtc**agc**tttcctccgaag**t**tg**a**acaaac
p50S342AR	ctgacttggaaact**gct**gaaccaaaacctttcctct	agaggaaaggttttggttc**agc**agtttccaagtcag
2nd reaction common reverse (Apa I)		**aca** *ggg ccc* tca tcc atg ctt cat ccc agc att aga ttt

Table 6
List of primers used to generate the p50 mutant constructs. The mutation for each construct is shown in bold and colour. The restriction enzyme sites used to clone the Flag and HA tagged mutant p50 into PCDNA3 are Hind III (aagctt) in the forward primer and Apa1 (gggccc) in the reverse primer. The start codon (**atg**) in the forward primer is highlighted in bold and orange.
